# Soil microbial community are more sensitive to ecological regions than cropping systems in alpine annual grassland of the Qinghai-Tibet Plateau

**DOI:** 10.3389/fmicb.2024.1345235

**Published:** 2024-03-15

**Authors:** Feng Luo, Wenbo Mi, Wenhui Liu, Xiang Ma, KaiQiang Liu, Zeliang Ju, Wen Li

**Affiliations:** ^1^Key Laboratory of Superior Forage Germplasm in the Qinghai-Tibetan Plateau, Qinghai Academy of Animal Husbandry and Veterinary Sciences, Qinghai University, Xining, China; ^2^Laboratory for Research and Utilization of Qinghai Tibet Plateau Germplasm Resources, Qinghai University, Xining, China

**Keywords:** cropping systems, ecological regions, forage mixtures, soil microbial diversity, soil microbial communities, Qinghai-Tibet Plateau

## Abstract

**Introduction:**

Modern agriculture emphasizes the design of cropping systems using ecological function and production services to achieve sustainability. The functional characteristics of plants (grasses vs. legumes) affect changes in soil microbial communities that drive agroecosystem services. Information on the relationship between legume-grass mixtures and soil microorganisms in different ecological zones guides decision-making toward eco-friendly and sustainable forage production. However, it is still poorly understood how cropping patterns affect soil microbial diversity in alpine grasslands and whether this effect varies with altitude.

**Methods:**

To fill this gap in knowledge, we conducted a field study to investigate the effects of growing oats (*Avena sativa* L.), forage peas (*Pisum sativum* L.), common cornflower (*Vicia sativa* L.), and fava beans (*Vicia faba* L.) in monocultures and mixtures on the soil microbial communities in three ecological zones of the high alpine zone.

**Results:**

We found that the fungal and bacterial community structure differed among the cropping patterns, particularly the community structure of the legume mixed cropping pattern was very different from that of monocropped oats. In all ecological zones, mixed cropping significantly (*p*  < 0.05) increased the α-diversity of the soil bacteria and fungi compared to oat monoculture. The α-diversity of the soil bacteria tended to increase with increasing elevation (MY [2,513 m] < HZ [2,661 m]  < GN [3,203 m]), while the opposite was true for fungi (except for the Chao1 index in HZ, which was the lowest). Mixed cropping increased the abundance of soil fungi and bacteria across ecological zones, particularly the relative abundances of *Nitrospira*, *Nitrososphaera*, *Phytophthora*, and *Acari*. Factors affecting the bacterial community structure included the cropping pattern, the ecological zone, water content, nitrate-nitrogen, nitrate reductase, and soil capacity, whereas factors affecting fungal community structure included the cropping pattern, the ecological zone, water content, pH, microbial biomass nitrogen, and catalase.

**Discussion:**

Our study highlights the variation in soil microbial communities among different in alpine ecological regions and their resilience to cropping systems. Our results also underscore that mixed legume planting is a sustainable and effective forage management practice for the Tibetan Plateau.

## 1 Introduction

Modern agriculture produces high yields through the extensive use of non-renewable energy and chemical inputs, a practice that is currently being called into question. Studies have shown that the costs of this model in terms of public health and environmental integrity are very high, hindering sustainable agricultural development (Tilman et al., 2002; Francis et al., 2016). As a result, the concept of “agroecology” has been developed (Gliessman, 1990; Wezel et al., 2014), which emphasizes the importance of designing cropping systems using ecosystem services and ecological principles to increase the sustainability and productivity of agroecosystems and reduce chemical inputs and non-renewable energy sources (Clergue et al., 2005; Faucon et al., 2017; Olounlade et al., 2017). To improve the ecological functioning of cropping systems, researchers have developed a range of practices based on agroecological guidelines, including mixed cropping, green manure, crop rotation, cover cropping, and intercropping (Wezel et al., 2014). Mixed cropping has great potential and is expected to significantly optimize cropping systems through plant diversification, particularly mixed grass and legume cropping systems (Crème et al., 2015; Zhao et al., 2015).

Legumes are often mixed with grasses in natural ecosystems, and legumes are often recognized as key ecologically efficient species (Altieri, 1999; Malézieux et al., 2009). The legumes and rhizomes in mixed cropping systems of grasses and legumes symbiotically fix atmospheric nitrogen and provide an additional source of nitrogen for the grasses. This nitrogen transfer mechanism frees the grass crop from nitrogen fertilizer limitations and limits “interspecific competition” due to “interspecific complementarity” (temporal, spatial, or chemical partitioning) (Hauggaard-Nielsen et al., 2008; Ye et al., 2020). Including legumes provides additional services to the cropping system, such as increased forage biomass (Tilman et al., 2002), nutrient quality (Tahir et al., 2023), resource utilization, and productivity (Loreau et al., 2001; Loreau and Hector, 2001). At the same time, polluting agro-ecosystems with inorganic fertilizers should be reduced (Heijden and Horton, 2009; Frankow-Lindberg and Dahlin, 2013). Moreover, mixed cropping also plays an important role in agroecosystem sustainability, i.e., utilizing the concept of plant complementarity to gain access to soil resources and facilitating processes occurring in the rhizosphere through positive plant–soil-microbe interactions (Duchene et al., 2017). In addition, mixed grasslands increase N utilization and mineralization by affecting the soil C/N balance. Mixed grasslands increase the input of organic matter and the population of beneficial soil microorganisms (Fornara and Tilman, 2008; Fornara et al., 2009; de Deyn et al., 2011). As a result, microbial enzyme activity and nutrient mineralization improve and yields increase (Sun et al., 2015). However, soil microbes are sensitive to environmental and crop responses (Brockett et al., 2012). Microbial activity and microbial biomass increase when a mixture of grass and legume crops is sown because the soil is so closely linked to the microbial community as it provides an ideal environment for bacteria, fungi, and a myriad of other organisms to survive (Luo et al., 2023). Plant diversity affects soil environmental conditions, with increased belowground biomass and root activity having significant effects on soil properties and soil secretions. Enhanced root activity and increased rhizome deposits provide nutrients to the microbial community (Wichern et al., 2007; Hinsinger et al., 2009), changing the structure of the microbial community (Hamilton and Frank, 2001; Wieland et al., 2001; Gabriele and Kornelia, 2010).

With an average altitude of 4,000 m and a cold climate, the Tibetan Plateau is known as the “Roof of the World.” The unique climatic conditions and geographic location created an alpine meadow ecosystem (Wei et al., 2014). The alpine meadows of the Tibetan Plateau are the largest natural grasslands in China, and they represent an ecological barrier as well as an important forage source (Hu et al., 2021). Livestock farming is the traditional and dominant local industry (Wei et al., 2017). However, in recent years, the grasslands have been degraded due to human activities, and climate change and other factors have reduced ecological service functions (Cao et al., 2019). Local livestock development is challenged, and planting ecologically sound pasture is essential to restore the grasslands and develop livestock (Dong et al., 2010). Therefore, natural nitrogen-fixing plants and leguminous forage have been introduced into the cropping system as an alternative to organic nitrogen fertilizer to increase forage production and mitigate negative environmental impacts. However, the functional characteristics of plants (grasses vs. legumes) also affect the amount and composition of resources in the soil microbial community that drive agroecosystem services, affecting their size, structure, and functioning (Zelles, 1999; Stephan and Schmid, 2000; Elling et al., 2016). It has been suggested that agricultural practices that increase microbial abundance and allow fungi to be predominant (expressed as the ratio of fungal biomass to bacterial biomass) will promote the accumulation of soil organic carbon (Spehn et al., 2000; Deyn et al., 2004). Increased diversity through intercropping improves below-ground productivity and provides heterogeneous carbon inputs, thereby increasing microbial abundance and activity in perennial forages (Peoples et al., 1995; Spehn et al., 2002; Wardle et al., 2006). However, changes in the microbial community composition may be affected by plant characteristics. Different conclusions have been reached from field-based studies on the effects of mixed grass-legume cropping on the microbial community. Some studies have clarified that grass-legume forage systems and inorganic nitrogen fertilizer affect the soil microbial community (Zhao et al., 2015). However, the alpine has many different ecological zones where many microclimates exist and there are large differences in altitude and climate. It is still unknown how monoculture and mixed cropping systems of leguminous forages and grasses affect the soil microbial community. Thus, we designed a large field study to assess how different ecological zones and cropping patterns affect the soil microbial community. This study will provide important information for managing of sustainable agriculture in the Tibetan Plateau region.

## 2 Materials and methods

### 2.1 Study site

**Figure 1:**
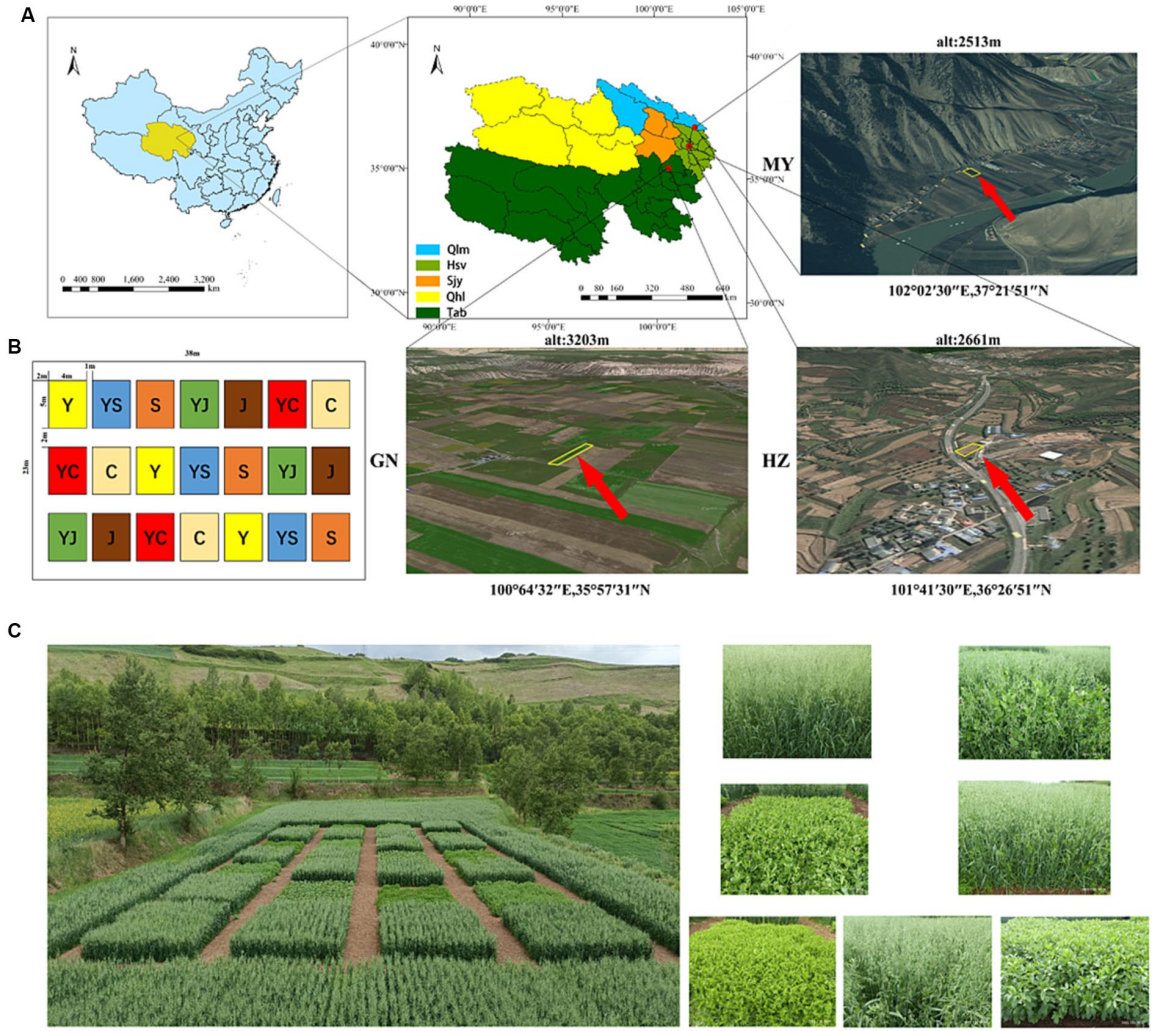
**(A)** Study area information. **(B)** Distribution of planting plots. **(C)** Field planting map. Y, oats unicast; YS, oats and forage peas mixed sowing; S, forage peas unicast; YJ, oats and common vetch mixed sowing; J, common vetch unicast; YC, oats and fava beans mixed sowing; C, fava beans unicast; HZ, Huangshui Valley; GN, Sanjiangyuan District; MY, Qilian Mountain Basin.

The experiment was executed in three ecological regions: HZ: Huangzhong County in Huangshui Valley. GN: Guinan County in Sanjiangyuan District. MY: Menyuan County in the Qilian Mountain Basin (Figure 1A). The climatic characteristics and soil physicochemical properties of each planting area were listed in Supplementary Figure S1 and Supplementary Table S1.

### 2.2 Experimental design

The trial employed a randomized block group design, comprising seven treatments (Figures 1B,C). Each treatment had three replication plots, resulting in a total of 21 plots. Experimental units consisted of plots with an area of 20 m^2^ (5 m × 4 m). The seeds were provided by the Qinghai Academy of Animal Husbandry and Veterinary Science (Table 1).

**Table 1:** Planting systems and sowing rate.

Treatments	Crop and species	Seeding quantity/kg·ha^−1^
Y	Oat (Qinghai 444)	225.00
S	Forage peas (Qingjian No. 1)	111.60
J	Common vetch (Ximu No. 324)	120.00
C	Fava bean (Qingcan No. 22)	165.30
Y/S	Oat/Forage peas	135.00/44.64
Y/J	Oat/Common vetch	157.50/36.00
Y/C	Oat/Fava bean	135.00/66.12

The HZ, MY, and GN test sites were sown basing on the local climate and sowing dates. Apply 75 kg·ha^−1^ of urea (46% N) and 150 kg·ha^−1^ of calcium superphosphate (12% P_2_O_5_) as basal fertilizer before sowing (Xiang, 2022). It has been shown that in the alpine region of Qinghai-Tibet, the optimum mixing ratios of oats with forage peas, oats and fava beans, and oats and common vetch are 6:4, 7:3, 6:4 (Ting-xu et al., 2022). The sowing amount is listed in Table 1. Field management was consistent with other crops. Manual weed control was carried out twice.

### 2.3 Sampling and measurements

The crops had different growth and harvesting periods due to the differences in climate and elevation among the three test sites. The HZ, MY, and GN test sites were harvested and soil samples were collected on September 6, September 20, and September 28, 2022, respectively.

#### 2.3.1 Soil sampling

We used the 5-point method to collect soil samples (0-10 cm) from each plot (Edwards, 2010). Each soil sample composite was homogenized, passed through a 2 mm sieve, and divided into two parts. A portion of the soil sample was air-dried before soil physical and chemical analysis. Another portion was air-dried before soil physical and chemical analysis. Another portion was stored at −80°C for subsequent microbial biomass analysis (Huhe et al., 2017).

#### 2.3.2 Soil physicochemical properties

Three samples were randomly selected from each treatment to determine the soil physicochemical properties. The specific measurement methods are shown in Appendix S1. Significant differences in the soil physicochemical properties were detected among the ecological zones with different cropping patterns (Supplementary Tables S2–S4).

#### 2.3.3 High throughput sequencing of the soil samples

We entrusted Wekemo Biotechnology Co., Ltd. (Guangzhou, China) to sequence the soil samples. Soil microbiome DNA was extracted from 0.25 g of soil using the HiPure Soil DNA Kit (Wekemo, Guangzhou, China). DNA concentration and quality were detected by NanoDrop 2000 (Thermo Fisher Scientific, Wilmington, DE, USA). The V3-V4 hypervariable region of the bacterial 16S rRNA gene was amplified using an ABI GeneAmp® 9,700 PCR Thermal Cycler (ABI, Foster City, CA, USA) with primer pairs 341F (5′-CCTACGGNGGCWGCAG-3′) and 806R (5′-GGACTACHVGGGTWTCTAAT-3′).

### 2.4 Statistical analysis

The differences in the soil physicochemical properties, enzyme activity, nitrogen fractions, bacterial and fungal diversity, and species abundance in the different cropping systems and cropping regions were examined by two-way analysis of variance followed by a multiple comparison (Duncan) analysis using SPSS 20.0 software (SPSS Inc., Chicago, IL, USA). A *p* < 0.05 was considered significant. Origin 2021 software (OriginLab, Northampton, MA, USA) was used to draw the graphs. The differences in the bacterial and fungal communities in the different cropping systems and cropping regions were visualized in the R software environment using nonmetric multidimensional scaling analysis (NMDS). Species composition histogram and Venn diagram were plotted using R language. A structural equation model (SEM) was conducted using R software. Statistical analyses and drawings were completed using R 4.3.1 for Windows and the “piecewiseSEM,” “psych,” “vegan” package.

## 3 Results

### 3.1 Microbial diversity

The Chao1, Observed, and Shannon indices of the soil bacteria increased with increasing altitude [MY (2,513 m) > HZ (2,661 m) > GN (3,203 m)]. Except the Shannon index, the Chao1 and Observed indices were significantly (*p* < 0.05) higher in GN and HZ than in MY (Table 2). However, the Chao1, Observed, and Shannon indices of soil fungi tended to decrease with increasing altitude (except for the lowest Chao1 in HZ) (Table 3). Mixed cropping significantly (*p* < 0.05) increased the Observed, Chao1, and Shannon indices of the soil bacteria and fungi compared to oats unicast in all ecological regions. The highest Chao1 index was observed in the ecological regions, except the highest altitude region (GN) where the cropping system was highest in mixed cropping. Notably, the α-diversity of soil fungi was highest in the YS cropping systems in HZ and MY, and in the YJ cropping system of GN (except Chao1).

**Table 2:** Alpha diversity of soil bacterial communities in cropping systems of different ecological regions.

Region	Diversity	Y	YS	S	YJ	J	YC	C
HZ	Chao1	2674.52 ± 81.23Ab	2846.96 ± 47.65Aa	2718.78 ± 52.36Aa	2823.81 ± 52.33Aa	2833.08 ± 25.66Ba	2624.09 ± 77.64Bb	2781.86 ± 35.32Ba
	Sobs	2639.00 ± 27.89Ab	2806.00 ± 32.14Ab	2686.00 ± 45.32Bab	2790.00 ± 23.60Ba	2803.00 ± 34.25Ba	2593.00 ± 87.36Bab	2952.00 ± 66.32Aa
	Shannon	10.20 ± 0.16Ab	10.39 ± 0.25Aa	10.29 ± 0.77Aa	10.36 ± 0.65Aa	10.43 ± 0.81Aa	10.28 ± 0.44Aa	10.31 ± 0.59Aa
GN	Chao1	2680.10 ± 33.32Ab	2931.28 ± 41.20Aa	2871.50 ± 44.66Aa	2984.214 ± 85.23Aa	2931.32 ± 74.21Aa	2966.90 ± 85.21Aa	2902.44 ± 49.31Aa
	Sobs	2638.00 ± 17.65Ab	2888.00 ± 21.54Aa	2836.03 ± 25.62Aa	2937.00 ± 24.58Aa	2904.00 ± 44.12Aa	2927.00 ± 58.62Aa	2737.00 ± 24.68Ba
	Shannon	10.36 ± 0.21Ab	10.40 ± 0.18Aa	10.42 ± 0.77Aa	10.50 ± 0.81Aa	10.42 ± 0.54Aa	10.47 ± 0.33Aa	10.43 ± 0.28Aa
MY	Chao1	2044.74 ± 24.31Bc	2691.76 ± 29.66Ba	2350.13 ± 33.65Bb	2916.38 ± 53.24Aa	2761.78 ± 26.90Ca	2937.61 ± 58.32Aa	2952.12 ± 85.24Aa
	Sobs	2018.00 ± 19.56Bc	2670.00 ± 56.24Ba	2330.00 ± 22.20Cb	2887.00 ± 32.64Aa	2733.00 ± 44.21Ca	2904.00 ± 25.31Aa	2917.00 ± 22.36Aa
	Shannon	8.57 ± 0.62Bc	9.24 ± 0.29Bb	8.02 ± 0.33Bc	10.41 ± 0.46Aa	10.33 ± 0.19Aa	10.56 ± 0.22Aa	10.57 ± 0.32Aa

Lowercase letters indicate significant differences in the same ecological regions for different planting patterns, uppercase letters represent significant differences in the same planting patterns for different ecological regions (*p* < 0.05) (The same as Table 3).

**Table 3:** Alpha diversity of soil fungi communities in cropping systems of different ecological regions.

Region	Diversity	Y	YS	S	YJ	J	YC	C
HZ	Chao1	420.31 ± 22.31Ab	566.23 ± 45.21Ba	529.61 ± 36.30Ba	417.03 ± 29.82Cb	412.01 ± 22.31Abc	372.02 ± 31.20Cc	457.41 ± 52.36Bb
	Sobs	7.38 ± 0.26Aa	7.47 ± 0.18Aa	6.98 ± 0.09Bb	6.85 ± 0.07Ab	6.33 ± 0.11Bc	6.92 ± 0.08Bb	6.96 ± 0.07Bb
	Shannon	7.30 ± 0.12Aa	7.47 ± 0.21Aa	7.10 ± 0.24Ba	6.84 ± 0.19Bab	6.78 ± 0.33Ab	6.93 ± 0.24Bab	6.95 ± 0.09Bab
GN	Chao1	365.80 ± 25.41 Bd	488.41 ± 33.60Cb	557.17 ± 41.21Ba	471.81 ± 25.64Ab	372.82 ± 23.61 Bd	511.24 ± 44.51Bab	436.43 ± 41.20Bc
	Sobs	6.25 ± 0.11Bc	6.62 ± 0.29Bb	7.03 ± 0.15Ba	7.11 ± 0.48Aa	6.72 ± 0.25Aab	7.04 ± 0.51Ba	6.63 ± 0.24Cb
	Shannon	6.25 ± 0.12Bc	6.62 ± 0.22Bb	77.03 ± 0.52Ba	7.09 ± 0.41Aa	6.68 ± 0.35Ab	7.03 ± 0.26Ba	6.61 ± 0.45Cb
MY	Chao1	281.23 ± 12.68Ce	66.25 ± 33.62Aa	616.80 ± 27.31Aa	446.61 ± 19.81Bc	399.43 ± 36.9Ad	575.04 ± 54.21Ab	553.62 ± 26.47Ab
	Sobs	6.17 ± 0.24Bc	7.73 ± 0.62Aa	7.41 ± 0.39Aab	7.03 ± 0.21Ab	6.81 ± 0.27Ab	7.61 ± 0.16Aa	7.44 ± 0.33Aab
	Shannon	6.36 ± 0.18Bc	7.73 ± 0.52Aa	7.41 ± 0.54Aab	7.03 ± 0.22Ab	6.81 ± 0.34Ab	7.62 ± 0.19Aa	7.43 ± 0.42Aab

### 3.2 Microbial community composition and relative abundance

**Figure 2:**
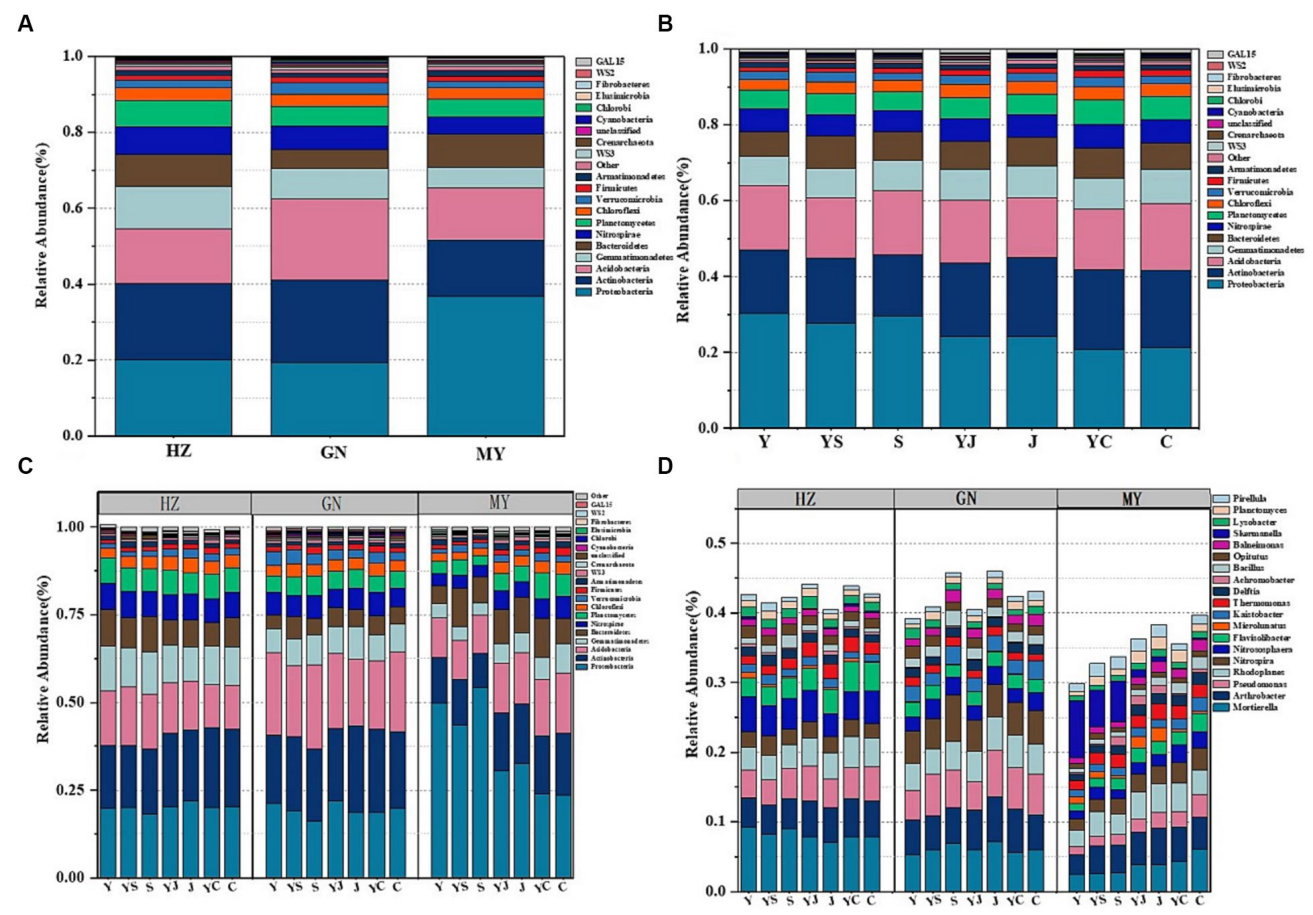
**(A)** Relative abundance at the bacterial phylum level in the different ecological regions (viewing the cropping systems. as a whole); **(B)** Relative abundance at the bacterial phylum level in the different cropping systems (considering the ecological regions as a whole); **(C)** Relative abundance of bacterial phyla in the different ecological regions under the different cropping systems; **(D)** Relative abundance of the bacterial genera in the different ecological regions under the different cropping patterns.

**Figure 3:**
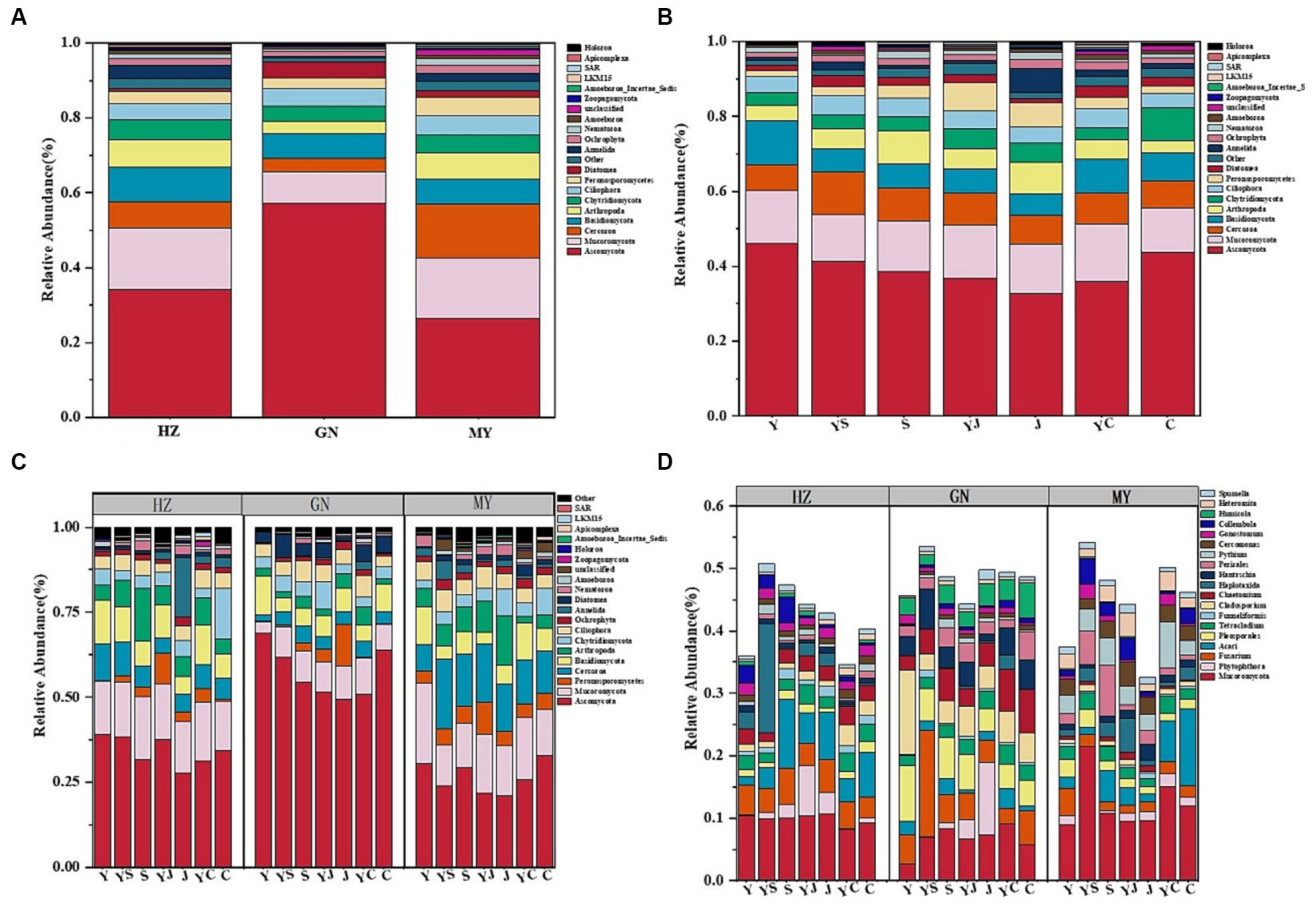
Relative abundance of fungal communities: **(A)** Relative abundance at the level of fungi phyla in different ecological regions (viewing the cropping systems. as a whole); **(B)** Relative abundance at the level of fungi phyla in different cropping systems (considering the ecological regions as a whole); **(C)** Relative abundance of fungi phyla in different ecological regions under different cropping systems; **(D)** Relative abundance of fungi genera in different ecological regions under different cropping patterns.

Among the three ecological regions and seven cropping systems, bacteria were dominated by *Proteobacteria*, *Actinobacteria*, *Acidobacteria*, *Gemmatimonadetes*, *Bacteroidetes*, and *Nitrospirae* (Figures 2A,B), and fungi were dominated by *Ascomycota*, *Mucoromycota*, *Cerozoa*, *Peronosporomy*, *Cercozoan*, *Basidiomycota*, and *Arthropoga* (Figures 3A,B), which accounted for 70% of the total number of sequence reads in the bacterial and fungal communities. The bacteria with the highest relative abundance were *Proteobacteria* among the bacteria at each level of the ecoregions (Figure 2A). The relative abundance of *Proteobacteria* decreased with elevation, followed by *Actinobacteria* and *Acidobacteria*, whose relative abundance increased with elevation, and fungi, whose relative abundance-altitude relationship was the opposite of that of bacteria (Figure 3A). The relative abundances of *Proteobacteria* and *Ascomycota* were highest in the Y cropping pattern, whereas mixed cropping reduced their relative abundances (Figures 2B, 3B). In contrast, the relative abundances of *Actinobacteria* and *Mucoromycota* were greater in the mixed cropping (YS, YJ, and YC) pattern than in the oat monoculture (Y), and mixed cropping increased their relative abundance.

**Figure 4:**
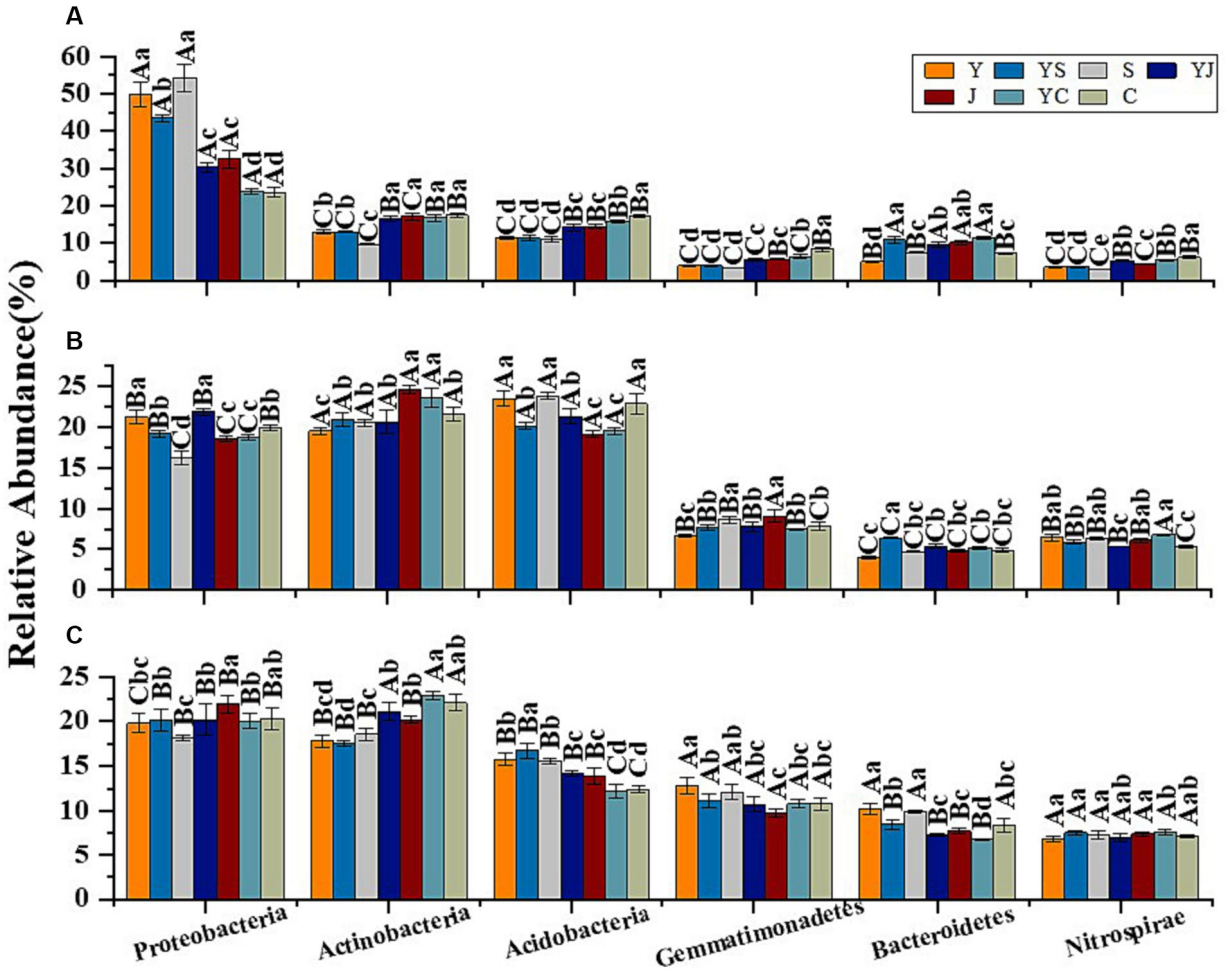
Dominant bacterial phyla in the different ecological regions and cropping patterns. **(A)** HZ; **(B)** GN; **(C)** MY.

**Figure 5:**
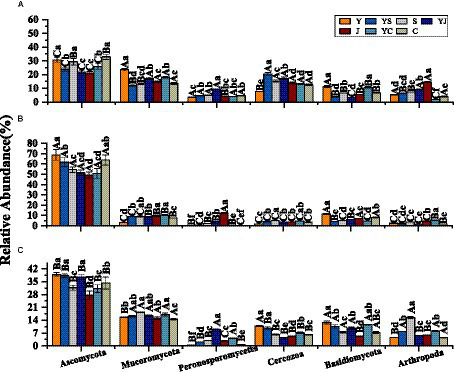
Dominant fungal phyla in different ecological regions cropping patterns. **(A)** HZ, Huangshui Valley; **(B)** GN, Sanjiangyuan District; **(C)** MY, Qilian Mountain Basin. The bars show the standard errors. Lowercase letters represent the significant difference within the same ecological regions under different cropping systems, while uppercase letters indicate the significant difference within different ecological regions under the same cropping systems. Significance was employed at 0.05.

The 20 bacteria and fungi commonly found at the phylum level in each planting pattern in the same ecoregion are shown in Figures 2C, 3C. The dominant bacteria in each ecoregion differed significantly (*p* < 0.05) compared with oat monoculture, while the low abundance of bacteria did not differ significantly; the same planting pattern of dominant bacteria differed significantly (*p* < 0.05) in different ecoregions (Figures 4, 5). The effects of cropping system on the relative abundance of bacteria at the soil gate level in the ecological regions are listed in Table 4. All had significant (*p* < 0.05) or highly significant (*p* < 0.01) effects, except for the interaction between the ecological regions and the cropping systems, which did not have a significant effect on the relative abundance of Actinobacteria and the cropping patterns on the relative abundances of *Acidobacteria*, *Gemmatimonadetes*, and *Bacteroidetes*. The effects of the cropping systems on the relative abundance of fungi at the soil gate level in the ecological regions are listed in Table 5. All had significant (*p* < 0.05) or highly significant (*p* < 0.01) effects, except for the ecological regions, which did not have a significant effect on the relative abundance of *Cercozoa* or *Arthropoda*, and cropping systems, which had a significant effect on the relative abundances of *Cercozoa* and *Basidiomycota*.

**Table 4:** Soil bacteria variance analysis of cropping systems in different ecological regions.

Source of variation	Proteobacteria	Actinobacteria	Acidobacteria	Gemmatimonadetes	Bacteroidetes	Nitrospirae
District	59.38***	42.95***	63.94***	206.88***	22.67***	88.33***
Treatment	3.73**	5.93***	0.58	2.05	1.06	2.87**
D * T	5.00***	0.98	2.84**	7.02***	2.27*	4.14***

*, ** and *** represent significant differences at the 0.05, 0.01 and 0.001 level, respectively.

**Table 5:** Soil fungi variance analysis of cropping systems in different ecological regions.

Source of variation	Ascomycota	Mucoromycota	Peronosporomycetes	Cercozoa	Basidiomycota	Arthropoda
District	208.84***	41.95***	291.10***	2.58	3.88*	0.33
Treatment	3.30**	3.49**	9.65***	1.18	0.67	3.03*
D * T	4.22***	2.42*	9.20***	2.97**	1.38	2.94**

*, ** and *** represent significant differences at the 0.05, 0.01 and 0.001 level, respectively.

The soil bacterial community at the genus level was dominated by *Ralstonia*, *Arthrobacter*, *Pseudomonas*, *Rhodoplanes*, *Nitrospira*, and *Nitrososphaera*. The soil fungal community at the genus level was dominated by *Mortierella*, *Phytophthora*, *Fusarium*, *Acari*, *Pleosporales*, and *Tetracladium*. The relative abundances of the dominant bacterial communities at the genus level were higher in HZ and GN than in MY. Additionally, the relative abundances of the bacterial and fungal communities at the genus level were also higher in the mixed cropping system than in the oat monocultured soil, particularly the relative abundances of *Nitrospira*, *Nitrososphaera*, *Phytophthora*, and *Acari* (Figures 2D, 3D). This finding suggests that the cropping patterns and ecological zones had significant effects on the dominant bacterial phyla and genera in the soils, whereas the effects on some sparse bacterial phyla and genera were not significant.

### 3.3 Abundance of microbial species

**Figure 6:**
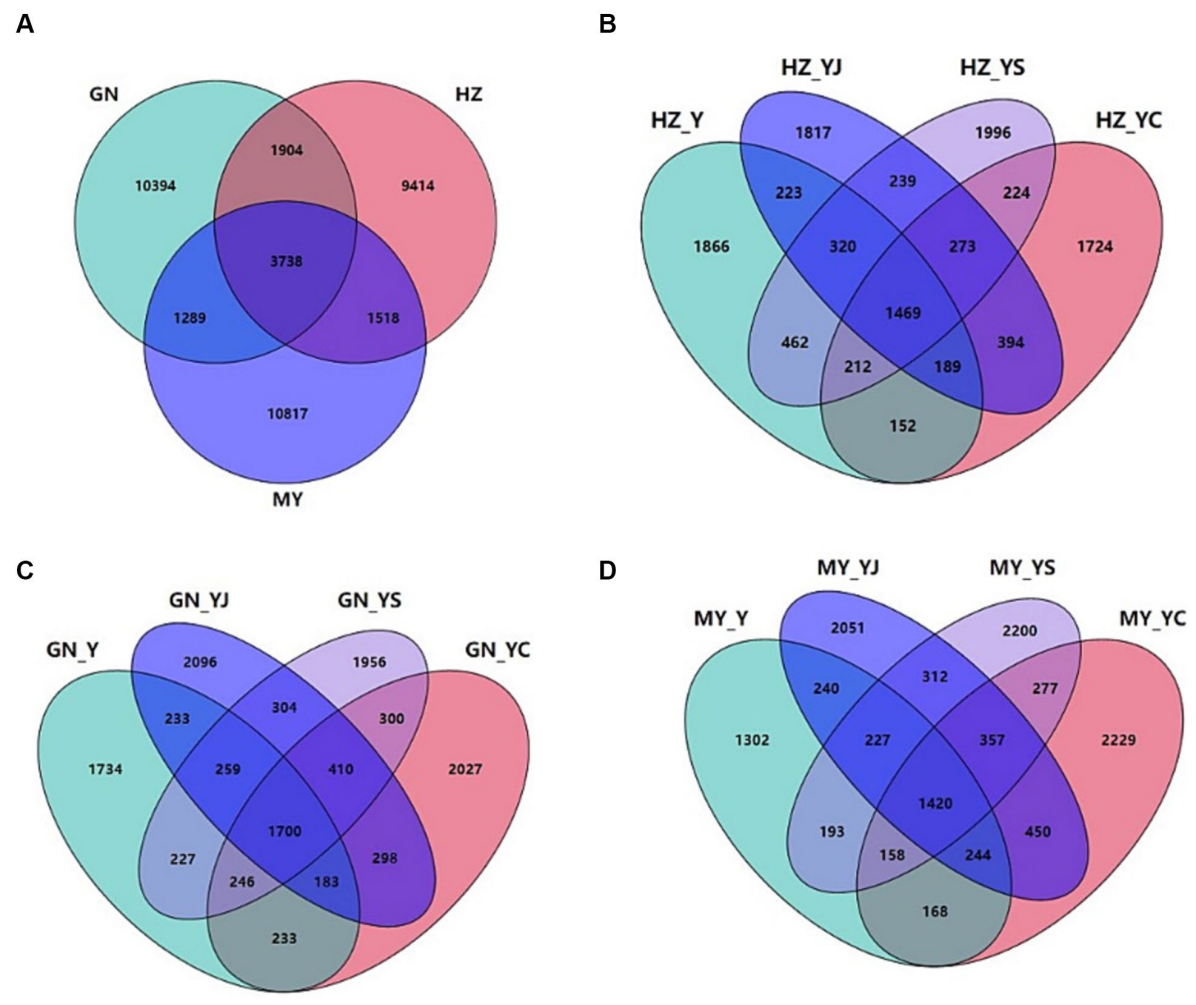
Bacterial Venn diagrams of the different ecological regions and different cropping systems. **(A)** Total number of OTUs in HZ, GN, and MY; **(B)** Number of OTUs in the cropping systems Y, YS, YJ, and YC in HZ; **(C)** Number of OTUs in the cropping systems Y, YS, YJ, and YC in GN; **(D)** Number of OTUs in the cropping systems Y, YS, YJ, and YC in MY.

**Figure 7:**
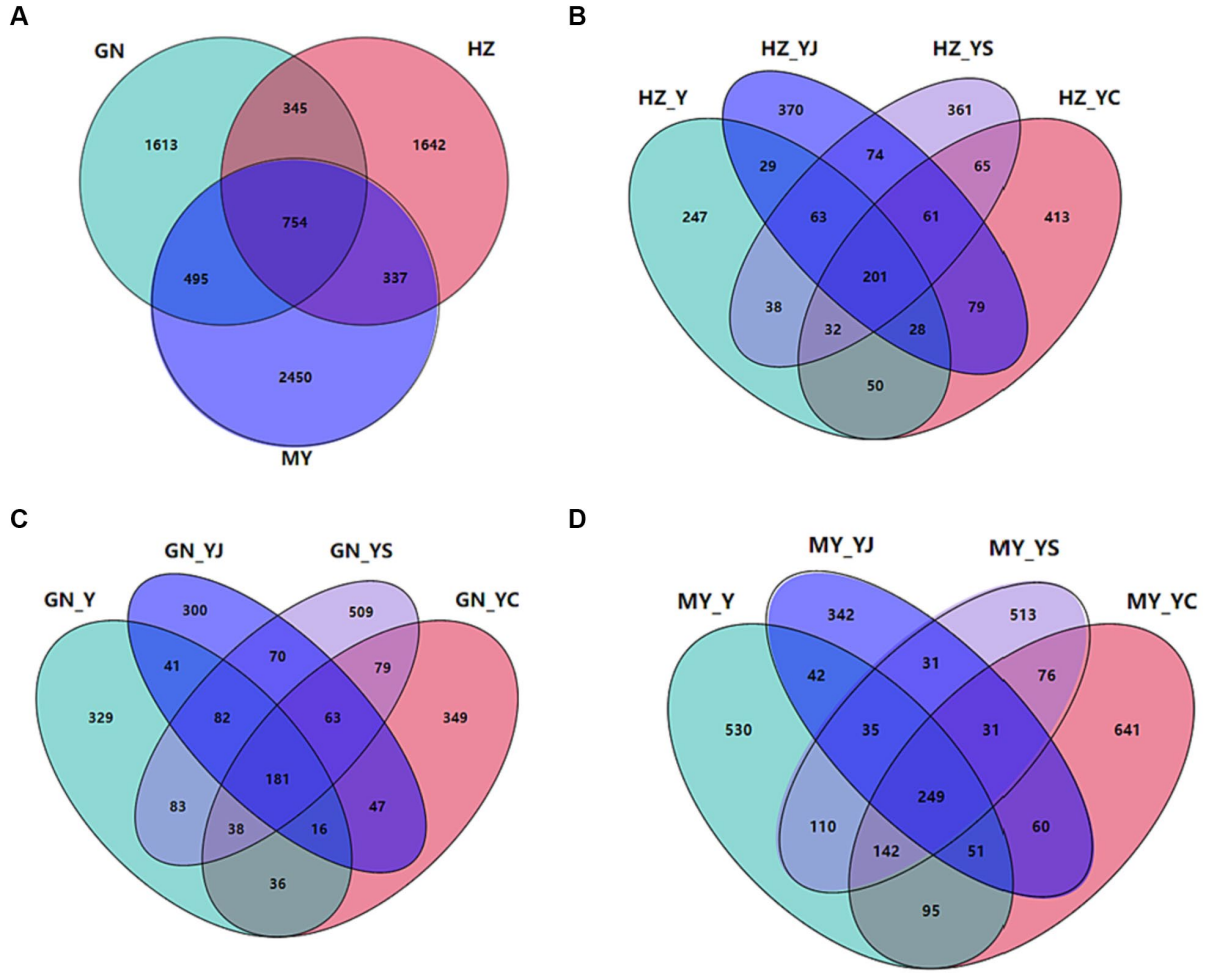
Fungal Venn diagrams of the different ecological regions and different cropping systems. **(A)** Total number of OTUs in HZ, GN, and MY; **(B)** Number of OTUs in the cropping systems Y, YS, YJ, and YC in HZ; **(C)** Number of OTUs in the cropping systems Y, YS, YJ, and YC in GN; **(D)** Number of OTUs in the cropping systems Y, YS, YJ, and YC in MY.

The sequencing results revealed a total of 39,074 bacterial OTUs and 7,636 fungal OTUs, of which 14,227 (36.41%) and 34,650 (88.68%) bacterial OTUs were annotated to the genus and phylum levels, respectively. Totals of 7,636 (1533%) and 6,920 (90.63%) OTUs were annotated at the fungal genus and phylum levels. Totals of 3,738 and 754 bacterial and fungal OTUs were shared by the HZ, GN, and MY soil samples respectively, with proportions of 9.57 and 9.88%. Of these, 9,414 and 1,642 bacterial and fungal OTUs were unique to HZ, 10,394 and 1,613 bacterial and fungal OTUs were unique to GN, and 10,817 and 2,450 bacterial and fungal OTUs were unique to MY (Figures 6A, 7A).

There were 1,223 or 7.29% HZ bacterial OTUs common to the soil samples of all of the cropping systems, whereas the bacterial OTUs specific to Y, YS, S, YJ, J, YC, and C included 1,548, 1,630, 1,530, 1,437, 1,739, 1,304, and 1,435, accounting for 3.96, 4.17, 3.92, 3.68, 4.45, 3.34, and 3.67%, respectively (Supplementary Figure S1A). A total of 273 bacterial OTUs were unique to the three mixed cropping systems except the oat monoculture (Figure 6B). A total of 1,374 or 7.93% of bacterial OTUs were common to the soil samples of all cropping systems in the GN region was, while the numbers of bacterial OTUs specific to Y, YS, S, YJ, J, YC, and C were 1,414, 1,549, 1,609, 1,686, 1,696, 1,626, and 1,533, accounting for 8.16, 8.94, 9.29, 9.73, 9.79, 9.39 and 8.85%, respectively (Supplementary Figure S1B). Among them, 410 bacterial OTUs were unique to the three mixed cropping systems (Figure 6C) and 1,044 (6.01%) bacterial OTUs were common to all cropping system soil samples in MY, whereas the bacterial OTUs specific to Y, YS, S, YJ, J, YC, and C included 1,045, 1,817, 1,666, 1,617, 1,523, 1,708, and 1,993, respectively, which accounted for 6.02, 1.04, 9.60, 9.31, 8.77, 9.84, and 11.48% (Supplementary Figure S1C). A total of 410 bacterial OTUs were unique to the three mixed cropping systems (Figure 6D).

A total of 144 or 4.68% fungal HZ OTUs were common to all cropping system soil samples, while 185, 259, 410, 284, 252, 289, and 240 bacterial OTUs were specific to Y, YS, S, YJ, J, YC, and C respectively, and accounted for 6.01, 8.41, 13.32, 9.23, 8.18, 9.39, and 7.80% (Supplementary Figure S2A). Sixty-one bacterial OTUs were unique to the three mixed cropping systems (Figure 7B). A total of 139 or 4.33% of bacterial OTUs were common to all cropping pattern soil samples in the GN region, while the bacterial OTUs specific to Y, YS, S, YJ, J, YC, and C were 258, 367, 322, 232, 215, 260, and 367, respectively, which accounted for 8.04, 11.44, 10.04, 7.23, 6.70, 8.11, and 11.44% (Supplementary Figure S2B). Among them, 63 bacterial OTUs were unique to the three mixed cropping systems, except the oat monoculture (Figure 7C). A total of 115 or 2.85% bacterial OTUs were common to the soil samples from all cropping systems in MY, whereas the bacterial OTUs specific to Y, YS, S, YJ, J, YC, and C included 446, 434, 401, 259, 196, 513, and 436 or 11.05, 10.75, 9.94, 6.42, 4.86, 12.71, and 10.80%, respectively (Supplementary Figure S2C) and 10.80% (Supplementary Figure S2C). Among them, 31 bacterial OTUs were unique to the three mixed cropping systems, except oat monoculture (Figure 7D).

### 3.4 Structure of the microbial communities

**Figure 8:**
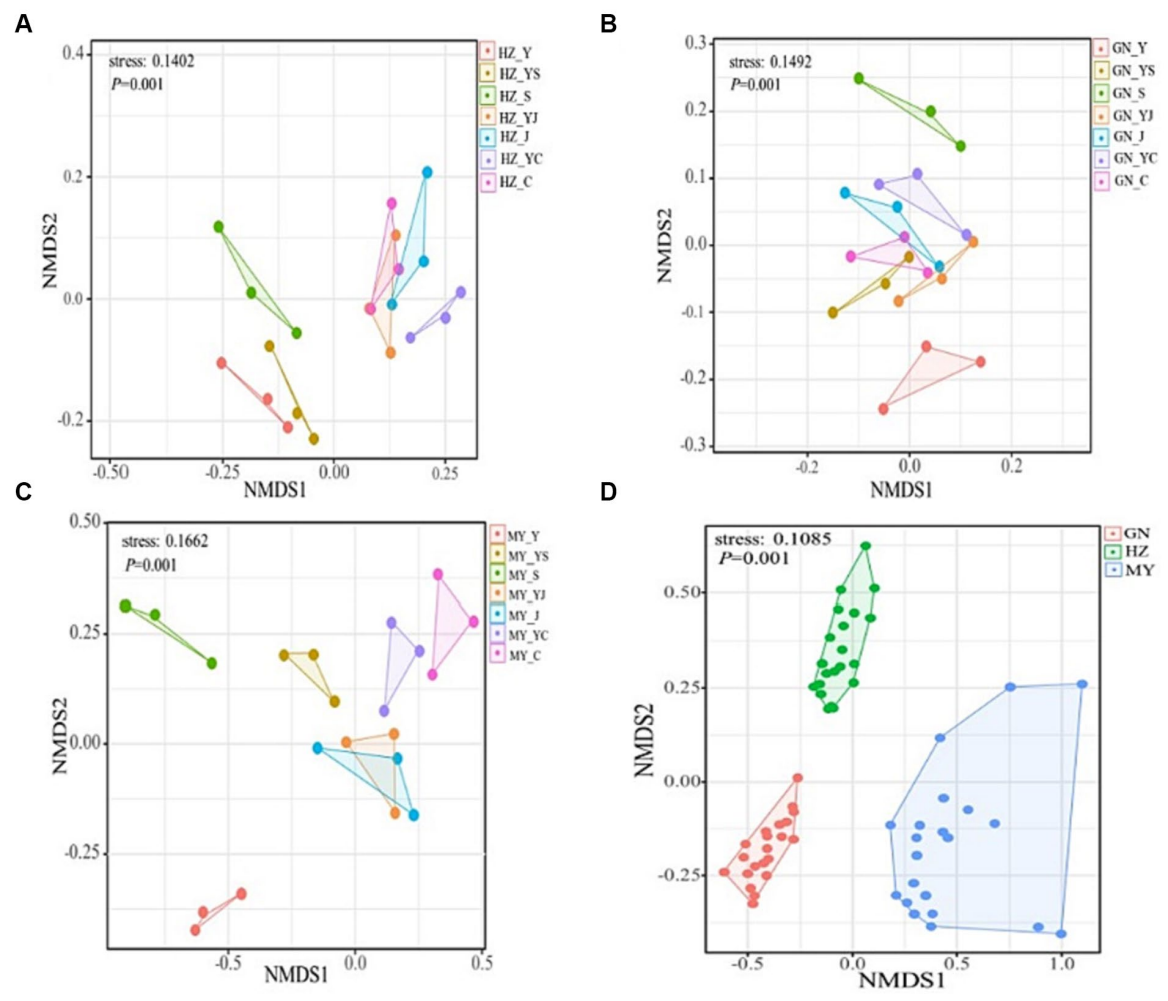
NMDS analysis of the bacterial OTU levels. **(A)** HZ. **(B)** GN. **(C)** MY. **(D)** Relative differences in the bacterial community composition in three ecological regions.

**Figure 9:**
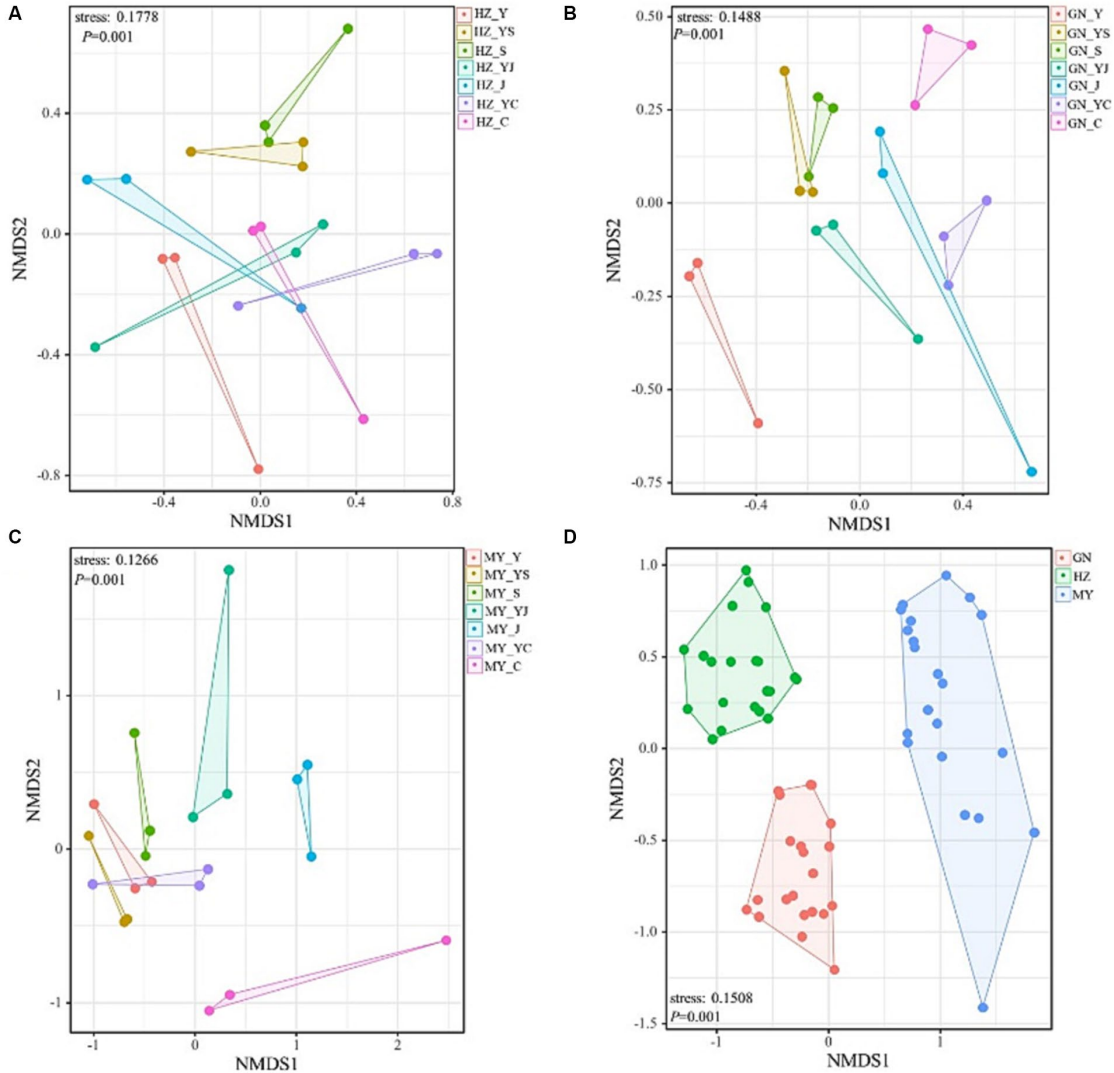
Non-metric multidimensional scaling (NMDS) analysis of the fungal OTU levels in the different ecological regions with different cropping systems. **(A)** Relative differences in the bacterial community composition in HZ. **(B)** Relative differences in the bacterial community composition in GN. **(C)** Relative differences in the bacterial community composition in MY.

The NMDS analysis showed that the fungi and bacteria had different group structures in different cropping systems of the same ecological region, particularly the oat monoculture community structure was significantly different from that of the legume mixed cropping system (Figures 8, 9A–C). No overlap was observed between the cropping systems in different ecological regions, indicating that the community structure of the cropping systems in different ecological regions differed significantly (Figures 8D, 9D).

**Figure 10:**
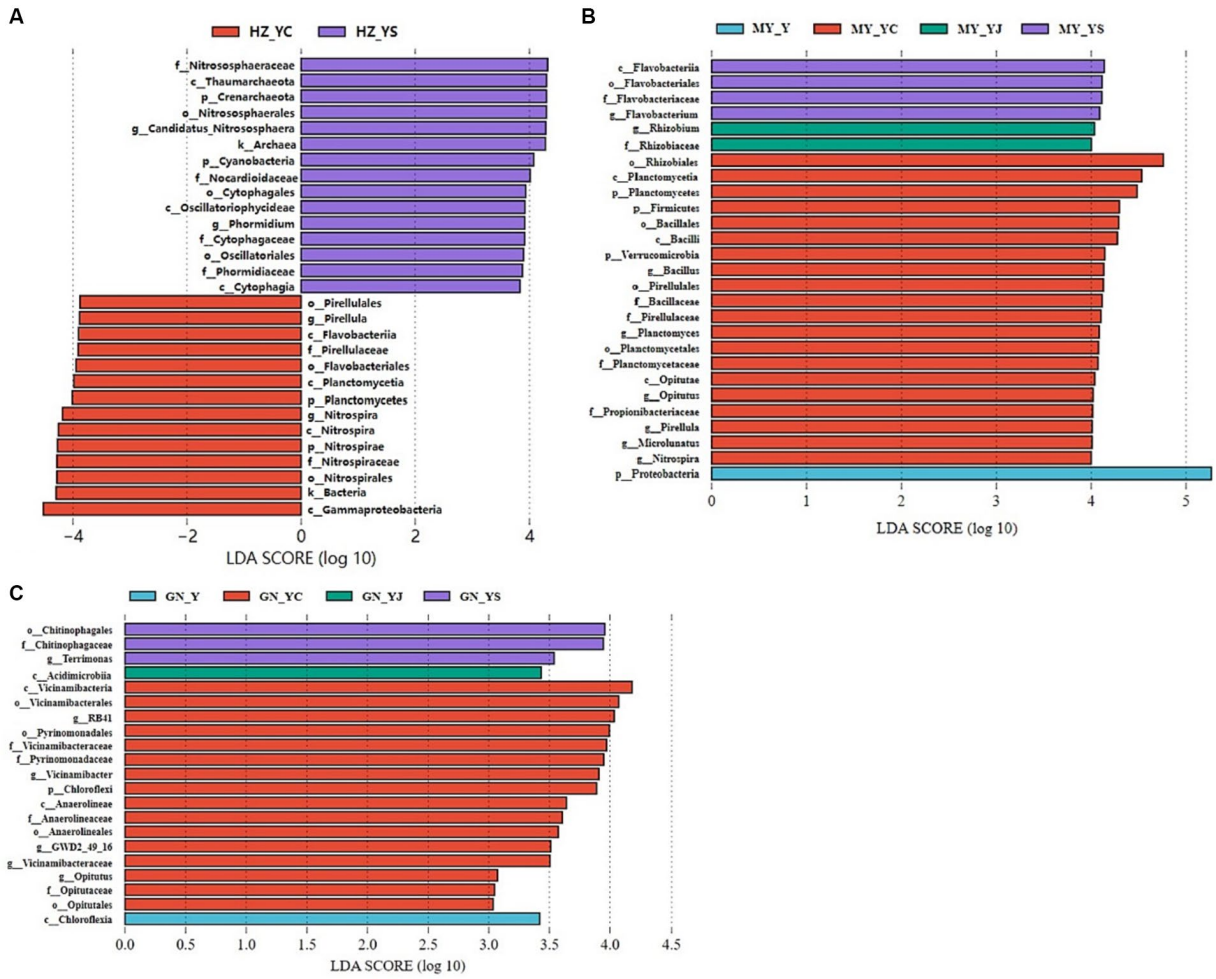
Changes in soil bacteria in the oat monoculture and the mixed cropping patterns across ecological zones analyzed by LEfSe. **(A)** HZ; **(B)** GN; **(C)** MY.

**Figure 11:**
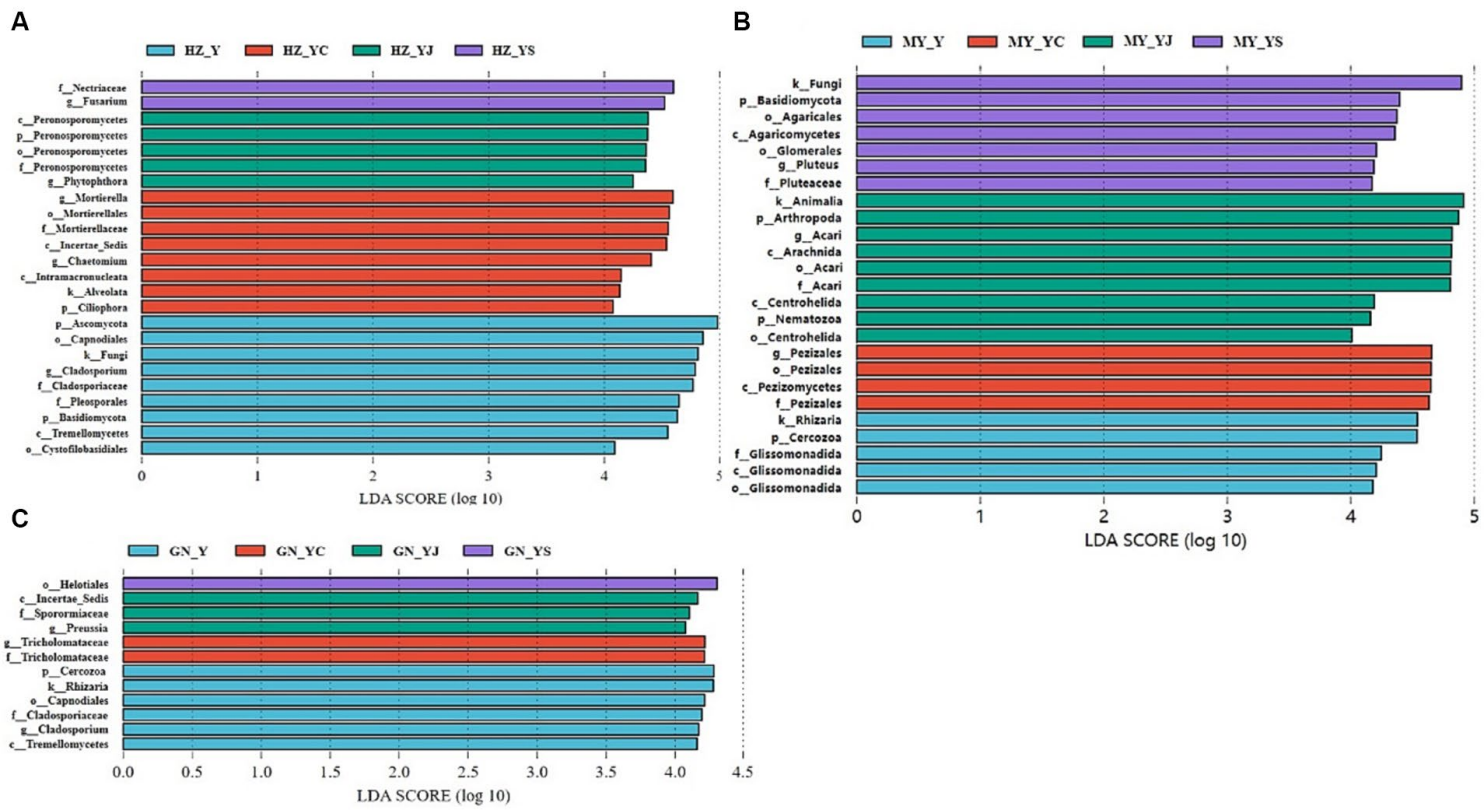
Changes in soil fungal in the oat monoculture and the mixed cropping patterns across ecological zones analyzed by LEfSe. **(A)** HZ; **(B)** GN; **(C)** MY.

Significant differences were detected in the fungal and bacterial community structure between the oat monoculture and the mixed cropping systems (Y, YS, YJ, and YC) in the same ecological region. Therefore, we further explored the differences in the microbial community structure among the four groups in each ecological region using LEfSe analysis (Figures 10, 11). We observed different bacterial biomarkers for the four cropping systems in the three ecological regions. The largest bacterial species in each region of the Y, YS, YJ, and YC cropping systems were *Rubellimicrobium*, *Nitrososphaeraceae*, *Cystobacterium*, and *Nitrospiraceae* (HZ) (Figure 10A); *Promicromonospora*, *Cytophagia*, *Lanctomyces*, and *Acidobacteria* (GN) (Figure 10B), and *Proteobacteria*, *Flavobacteriia*, *Rhizobium*, and *Rhizobiales* (MY) (Figure 10C). In addition, more *Nitrospiracea*e and *Rhizobium* were observed in the YS and YC mixed cropping systems in the HZ and MY regions, while relatively fewer were detected in the GN region. Notably, the YJ cropping system had a lower number of bacteria than the other two.

The fungal biomarkers in the four cropping systems were significantly different in each of the ecological regions. The largest fungal species in each region of the Y, YS, YJ and YC cropping patterns were Ascomycota, Nectriaceae, Peronosporomycetes, and Mortierella (HZ) (Figure 11A); Cercozoa, Helotiales, Incertae_Sedis, andTricholomataceae (GN) (Figure 11B), and Rhizaria, Basidiomycota,Animalia, and Pezizales (MY) (Figure 11C). Additionally, the fungalcommunity structure of the same cropping system was significantlydifferent in different ecological regions. Most importantly, the oatmonocrop in the GN region had significantly more fungi than thethree mixed cropping systems.

### 3.5 Key drivers of microbial community change

**Figure 12:**
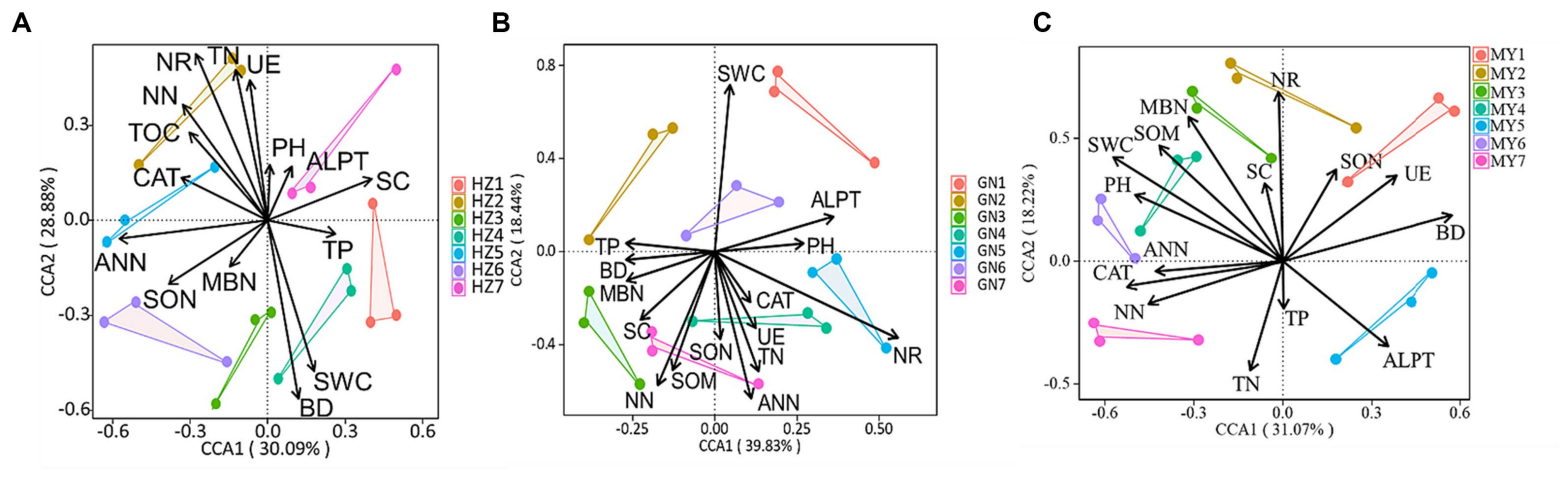
Typical correspondence analysis using pooled bacterial community data with the soil environmental factors (arrows). Values on axes 1 and 2 are the percentages that can be interpreted for the corresponding axes. **(A)** HZ; **(B)** GN; **(C)** MY.

**Figure 13:**
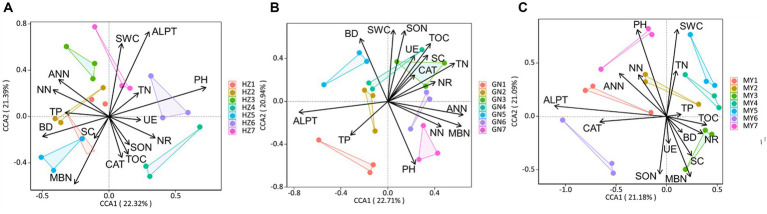
Typical correspondence analysis using pooled fungal community data with the soil environmental factors (arrows). Values on axes 1 and 2 are the percentages that can be interpreted for the corresponding axes. **(A)** HZ; **(B)** GN; **(C)** MY.

**Figure 14:**
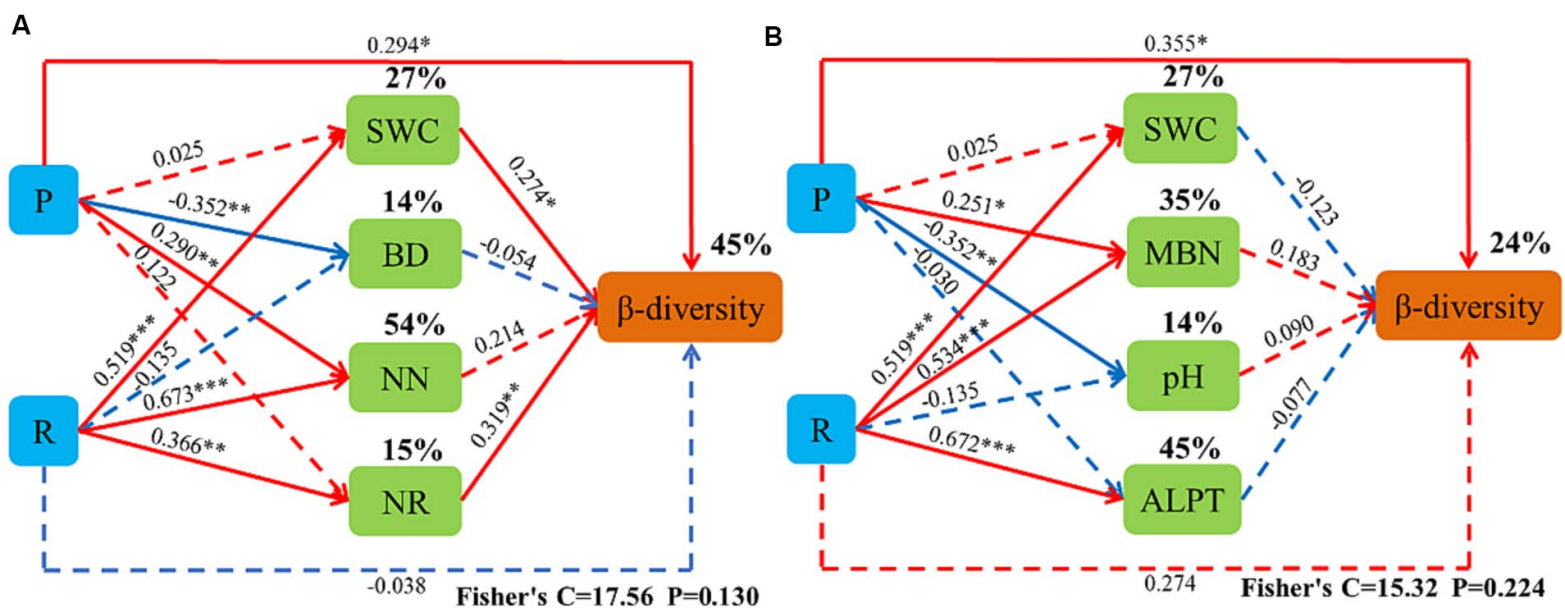
Piecewise structural equation model (SEM) describing the effect of the cropping pattern on soil bacterial **(A)** and fungal **(B)** β-diversity in the different ecoregions. P, cropping pattern; R, ecoregion; SWC, soil water content; BD, soil bulk density; NN, soil ammonium-nitrogen; ANN, soil nitrate-nitrogen; MBN, soil microbial nitrogen; pH, Pondus Hydrogenii; ALPT, soil catalase.

The canonical correspondence analysis (CCA) showed that the bacterial community composition was correlated (*p* < 0.05) with soil total nitrogen (TN), ammonium-nitrogen (ANN), nitrate-nitrogen (NN), bulk density (BD), soil water content (SWC), soil organic matter (SOM), nitrate reductase (NR) and soluble sugars (SC) (Figure 12). Fungal community composition was correlated (*p* < 0.05) with SWC, soil pH (pH), TN, NN, ANN, soluble organic nitrogen (SON), and catalase (CAT) (Figure 13). Variables that significantly affected the composition of the CCA microbial communities were selected as predictors to generate slice SEMs to identify the key drivers of soil microbial β-diversity. The bacterial pathway analysis showed that the cropping system (P), SWC, and NR had significant positive effects on soil bacterial β-diversity, and P indirectly affected soil bacterial β-diversity by significantly promoting NN and suppressing BD (Figure 14A). The cropping regions (R) indirectly affected β-diversity by significantly contributing to SWC, NN, and NR. The pathway analyses for fungi showed that P had a significant positive effect on soil fungal β-diversity, and that P indirectly affected soil fungal β-diversity by significantly promoting MBN and suppressing pH. R indirectly affected fungal β-diversity by significantly promoting SWC, MBN, and ALPT (Figure 14B).

## 4 Discussion

### 4.1 Effect of the cropping pattern on the soil microbial communities

Soil microorganisms are indicators of soil health and are involved in nutrient cycling (Crecchio et al., 2004) and transformation processes that are sensitive to changes in the soil (Song-Juan et al., 2015). Mixed cropping directly affects the soil microbial community structure by altering soil nutrient availability (Zhao et al., 2015; Ye et al., 2020). In this study, soil bacterial and fungal diversity and community structure varied among the cropping patterns. Incorporating legume forage increased the α-diversity of the soil fungi and bacteria compared to the oat monoculture, and the cropping pattern with the highest Observed and Shannon indices of soil bacteria and fungi in all ecological zones was the legume-oat mix. Many studies have shown that nitrogen-fixing plants have a greater effect on soil microorganisms than graminaceous plants (both C3-and C4-graminaceous plants) (Stephan and Schmid, 2000; Deyn et al., 2004). Soil fertility is improved significantly, nutrient cycling and nitrogen mineralization in the soil are enhanced, and organic matter inputs and populations of beneficial soil microorganisms increase due to the characteristic rhizomatous and nitrogen-fixing capacity of legumes. On the other hand, legume apoplasts are nitrogen-rich and readily decomposed by bacteria and fungi (Wardle et al., 2006; de Deyn et al., 2011; Zhao, 2014), which increase the activity of fungal-mediated decomposition pathways and accelerate resource inputs of relatively difficult-to-decompose substrates (e.g., cellulose, hemicellulose, tannins, and lignin) to fungal-mediated decomposition pathways. In addition, an increase in legume-specific nitrogen-fixing microorganisms shifts the soil microbial community from one dominant species to another (Ben-Chuan et al., 2022) by increasing soil microbial abundance (Dommergues, 1987; Murali et al., 2012). At the same time, legume-induced changes in soil microorganisms control higher trophic level organisms, such as micropredators, omnivores, and predators, from the bottom up through the food chain (Zhao et al., 2013; Zhao, 2014).

In this study, the relative abundances of the dominant bacteria Proteobacteria and the fungus Ascomycota were the highest in the Y cropping pattern at the gate level of each cropping pattern, and mixed cropping reduced the relative abundances of these two bacteria. In contrast, the relative abundances of the bacterium Actinobacteria and the fungus Mucoromycota were greater for the mixed cropping (YS, YJ, and YC) patterns than the oat monocrop (Y), and mixed cropping increased their relative abundance. This may be due to the ability of Actinobacteria and Mucoromycota to decompose organic matter, such as cellulose and lignin, which promotes the formation of soil aggregate structure and improves soil quality. In contrast, mixed cropping increases the diversity of apoplastic material, which is beneficial for microbial activity, and, therefore, actinomycetes increases in mixed grass and legume soils (Li et al., 2020). Secondly, the rhizomatous action of legumes improves soil N utilization and cycling capacity and increases the abundance of microorganisms associated with soil N cycling (Wen et al., 2003; Erohin, 2015). In addition, the relative abundance of bacterial and fungal communities at the genus level was higher in the mixed-seeded soils than in the oat monoculture soils, particularly for *Nitrospira*, *Nitrososphaera*, *Phytophthora*, and *Acari*. *Nitrospira* are important drivers of nitrification in the soil and plays an important role in the nitrogen cycle, effectively contributing to nitrogen use in the soil (Nosheen and Bano, 2011). The soil bacterial and fungal community structures varied among cropping patterns, particularly for the oat monocrop, which was very different from that of the legume mixtures. This is because the microbial community structure is primarily driven by plant diversity and dominant strains (Liu et al., 2023). Although mixed seeding improve plant diversity, different soil environments and plant characteristics produce different ecological niches, which can affect specific soil microbial communities (Khuroo et al., 2021). Differences in the amount and composition of root secretions of different species lead to changes in soil microbial metabolism, which affects the composition of soil microorganisms (Nayyar et al., 2010).

### 4.2 Influence of the ecoregions on the soil microbial communities

Soil microorganisms respond sensitively to small changes in environmental factors that alter their community composition and structure (Janssen, 2006; Alfonso Molina et al., 2010). Factors, such as altitude (Yang et al., 2014), soil temperature, humidity (Angel et al., 2010; Nielsen et al., 2010; Zhou et al., 2016), soil physicochemical properties (Fierer and Jackson, 2006), and vegetation type (Knelman et al., 2012) cause changes in the diversity and structure of the soil microbial communities. Elevation is one of the most important environmental factors that indirectly affects soil microbial community characteristics by regulating the microclimate of the local area (Shen et al., 2020). Our study area was located in three different ecoregions of the Tibetan Plateau with large differences in elevation and climate. We showed that soil bacterial and fungal diversity and community structure varied significantly across ecological zones, which was consistent with similar studies on the Tibetan Plateau (Wang et al., 2015; Zhu et al., 2020). Alpha diversity among the soil bacteria tended to increase with increasing elevation (MY [2,513 m] < HZ [2,661 m] < GN [3,203 m]), while the opposite was true for fungi. This was contrary to the findings of Zhu et al. (2020) who concluded that soil bacterial diversity was higher at lower elevations and suggested that this pattern was related to higher vegetation diversity at lower elevations. This result may have been due to differences in climatic conditions within specific altitudinal zones (Press, 2009), differences in sensitivity of soil bacteria and fungi to the environment, or differences in the initial microbial communities in different ecological zones (Luo et al., 2023). In addition, soil fungal and bacterial α-diversity in the same cropping pattern differed significantly across ecological zones. Among them, the α-diversity of soil fungi was highest in HZ and MY of the YS cropping pattern and in GN of the YJ cropping pattern. This may be because temperature and rainfall decreased with increasing altitude, while crops adapted differently to the environment and soil microbes responded differently to the cropping pattern. Meanwhile, differences in nitrogen fixation capacity of leguminous crops in different ecological regions also led to differences in soil nitrogen utilization, indirectly affecting the diversity of the soil microorganisms (Kennedy and Tchan, 1992; Rahman, 2013). Among the bacteria at the phylum level in the planting systems of each ecoregion, the bacteria with the highest relative abundance were Proteobacteria, whose relative abundance decreased with elevation, followed by Actinobacteria and Acidobacteria, whose relative abundance increased with elevation, and fungi, whose relative abundance and elevation relationships were the opposite of those of bacteria. This is similar to the findings of a previous study (Congcong et al., 2013). The phylum Proteobacteria was the most abundant among the flora of the different ecological zones studied, followed by Actinobacteria, which agreed with the results of a previous study (Jing et al., 2018). High environmental adaptation, rapid reproduction, and substrate uptake of Proteobacteria are the key reasons for their dominance in the soils of different ecological zones in the alpine (Salcher et al., 2010). The results of this study show that more Nitrospiraceae and *Rhizobium* were observed in the YS and YC mixed cropping pattern in the HZ and MY, while relatively less was detected in GN. The current findings confirm that bacterial abundance and uniformity associated with soil nitrification and nitrogen fixation decrease with increasing altitude (Wang et al., 2019). This may be because the relatively favorable temperatures and humidity at low and middle elevations maintain high soil microbial and plant diversity, whereas the cold and humid climate at higher elevations is not conducive to the growth and propagation of slow-growing *Rhizobium* spp. (Brown, 2001). However, nitrogen-fixing bacteria directly improve soil nutrient availability, favor plant growth, and enhance vegetation productivity and diversity, which, in turn, enhances the abundance of functionally specific soil microbial communities (Hartmann et al., 2009; Paul, 2016).

### 4.3 Key factors affecting the microbial communities

Cropping patterns and ecological zones affect soil microbial communities either directly through changes in the belowground soil environment or indirectly through effects on aboveground plant diversity. Soil environmental factors and soil microbial community structure are correlated (Liu et al., 2017; Tahir et al., 2023). Soil nutrient content, BD, soil moisture content, enzyme activity, and pH are the main drivers of soil microbial community composition (Sloey and Hester, 2016). Previous studies have shown that soil SOM, pH, and bulk weight help explain the changes in soil microbial composition observed in this study, while changes in soil quick nutrients (N-NO_3_, AP, and AK) have a significant effect on bacterial diversity and abundance (Li et al., 2022). Incorporating fresh organic matter from legumes increases the decomposition rate of SOM, which stimulates the activities of the soil microbial communities involved in stabilizing SOM mineralization (Fontaine et al., 2003; Blagodatskaya and Kuzyakov, 2008; Bernard et al., 2009). This observation suggests that incorporating legumes into the cropping system would enhance bacterial mineralization of SOM and benefit the crops (Khatiwada et al., 2020; Ladha et al., 2022; Tahir et al., 2022). The results of this study show that cropping patterns (P), SWC and NR had significant positive effects on soil bacterial β-diversity, and P indirectly affected soil bacterial β-diversity by significantly promoting NN and suppressing BD. The ecoregion (R) indirectly affected β-diversity by significantly contributing to SWC, NN, and NR. P had a significant positive effect on soil fungal β-diversity. P indirectly affected soil fungal β-diversity by significantly promoting MBN and suppressing pH. R indirectly affected soil fungal β-diversity by significantly promoting SWC, MBN, and ALPT. The reasons for this may be (1) because mixed cropping increases species diversity with complementary ecological niche advantages, and the unique rhizomatous and nitrogen-fixing capacity of legumes significantly improves soil fertility, enhances nutrient cycling and nitrogen mineralization in the soil, and increases the input of organic matter and the number of beneficial soil microorganisms (Ye et al., 2020; Tahir et al., 2023). (2) Legume apoplasts are nitrogen-rich and readily decomposed by microorganisms, increasing the activity of microbial-mediated decomposition pathways, which accelerates resource inputs of relatively difficult-to-decompose substrates (e.g., cellulose, hemicellulose, tannins, and lignin) to fungal-mediated decomposition pathways (Zhao et al., 2015). (3) Feedback regulation by soil microorganisms. Nitrogen-fixing bacteria produced by legume nodules shift the soil microbial community from one dominant species to another (Ben-Chuan et al., 2022) increasing soil microbial abundance (Kuypers et al., 2018). (4) Soil bacteria and fungi differ in their sensitivity to the environment. Due to the different adaptability of crops to the environment, differences in the biological nitrogen fixation capacity of leguminous crops in different ecological regions leads to differences in soil nitrogen cycling and utilization, indirectly affecting the diversity of soil microorganisms (Luo et al., 2023).

## 5 Conclusion

The composition of the soil microbial community was influenced by the cropping system (grass monoculture, legume monoculture, or a mixture of grasses and legumes), particularly in the high-elevation (GN) and mid-elevation (HZ) ecoregions. Our results show that fungi and bacteria had different community structures in different cropping patterns, and in particular, the community structure of the legume mixed cropping pattern was very different from that of oat monocropping. The relative abundance of dominant bacterial and fungal genera was higher in the mixed-seeded soil than in the oat monoculture soil. In addition, there were differences in the response of each cropping pattern to different ecological zones, with decreases in bacterial diversity at high altitude but increases in fungal diversity, while soil microbial diversity and abundance were higher in the mixed cropping system in all ecological regions. Our study showed the impact of altitude factors on Legume-grass mixtures grasslands, highlighting to consider this information to better understand the interaction between soil microorganisms and grasses in alpine grassland ecosystems.

## Data availability statement

The original contributions presented in the study are included in the article/Supplementary material, further inquiries can be directed to the corresponding author.

## Author contributions

FL: Data curation, Formal analysis, Software, Writing – original draft, Writing – review & editing. WM: Data curation, Software, Writing – review & editing. WhL: Funding acquisition, Methodology, Writing – review & editing. XM: Data curation, Writing – review & editing. KL: Software, Writing – review & editing. ZJ: Software, Writing – review & editing. WL: Software, Writing – review & editing.
