# Supplementary material for: Soil microbial community are more sensitive to ecological regions than cropping systems in alpine annual grassland of the Qinghai-Tibet Plateau
Source: Front Microbiol. 2024 Mar 15;15:1345235. doi: 10.3389/fmicb.2024.1345235 (PMC10978683; doi:10.3389/fmicb.2024.1345235)
Supplement: Supplementary file 1 [file Data_Sheet_1.docx]

2.3.2 Soil physical and chemical properties

The soil pH was measured with a digital pH meter (Seven2Go, Mettler-Toledo Instruments Co., Ltd, Shanghai, China) in a 1:2.5 soil/water suspension after shaking at 250 rpm for 5 min. The soil physicochemical properties were determined following the “Soil Sampling and Methods of Analysis” (Crepin and Johnson, 1993). The soil bulk density (BD) was measured with the cutting ring (100cm^3^) method. Total soil porosity (TP) was obtained by coefficient conversion. Soil organic matter (SOM) was measured using the K_2_Cr_2_O_7_ redox titration method. Total nitrogen (TN) in the soil was determined by Kjeldahl digestion. Soil ammonium nitrogen (ANN) was determined by the indophenol blue colorimetric method. Nitrate nitrogen (NN) in the soil was assessed using the phenol disulfonic acid colorimetric method. The soil SON was extracted by hot water (70 °C) using the modified methods described by Chen (Chen et al., 2005). Briefly, 10 g (dry weight equivalent) of fresh soil was mixed with 40 mL of distilled water in a Falcon tube, and the tube was placed in a hot water bath for 18 h at 70 °C. The tubes were subsequently shaken for 5 min on an end-to-end shaker and filtered through Whatman 42 paper followed by a 0.45 mm filter membrane. The concentrations of NH_4_^+^-N, NO_3_^−^-N and NO_2_^−^-N in the extracts were measured using a LACHAT QuickChem Au-tomated Ion Analyser (QuikChem Method 10-107-06-04-D for NH_4_^+^ -N and Quik Chem Method 12-107-04-1-B for NO_3_^−^-N). The soluble inor-ganic N (SIN) was calculated as the sum of the NH_4_^+^-N, NO_2_^−^-N and NO_3_^−^-N in the extracts. The total soluble N (TSN) in the extracts was an-alzyed by the high temperature catalytic oxidation method using a SHIMADZU TOC analyzer (fitted with a TN unit) as described by Chen (Chen et al., 2005). The SON was calculated as the difference between the TSN and SIN. Soil microbial biomass nitrogen (MBN) was determined by leaching using chloroform fumigation (Joergensen, 1996).2.3.3 Soil enzyme activities

The nitrate reductase (NR) activity was measured following the method described by (Abdelmagid and Tabatabai, 1987) using KNO_3_ as the substrate. It was calculated after NO_3_^−^ reduction following 24^-^h incubation at 25 ^◦^C. The urease (UE) activity was assessed by incubating 5 g of the fresh soil sample for 2 h at 37 ^◦^C with 2.5 mL of a 0.08 M urea solution. The NH_4_^+^ content was determined using a spectrophotometer at 690 nm (Kandeler and Gerber, 1988). The soil sucrase (SC) activity was evaluated by determining the glucose discharge from an 8% sucrose solution following 24 h of incubation at 37 °C (Chen et al., 2010). The alkaline protease (ALPT) activity was analyzed using the ninhydrin colorimetric method (Liu et al., 2015). The potassium permanganate titration method was used to assess the catalase (CAT) activity (Wang et al., 2021).


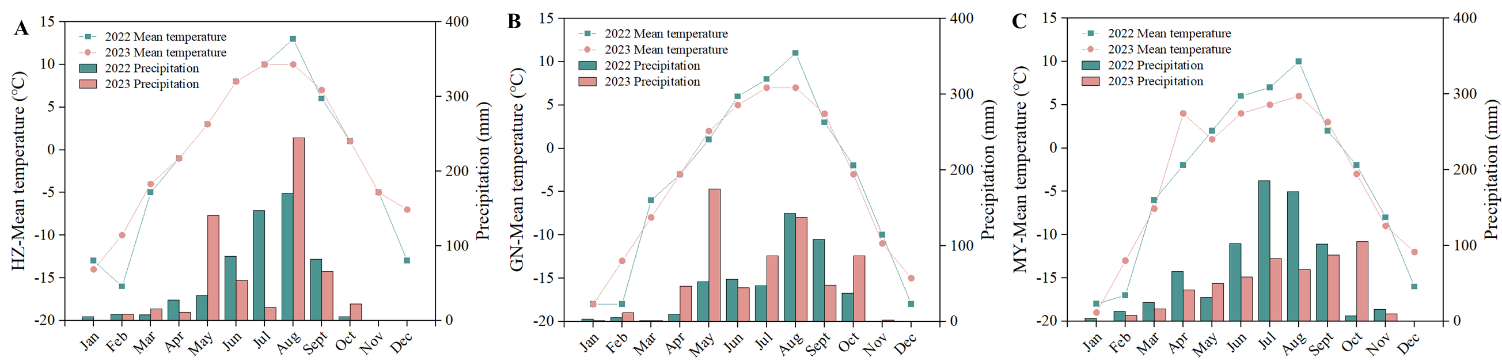


Supplementary Figure 1: Rainfall and temperature in the study area.

(A), HZ; (B), GN; (C), MY.

Supplementary TABLE 1 Soil physicochemistry of the study site

|  | Ecological region profile | | | |
| --- | --- | --- | --- | --- |
| Basic information | | HZ | GN | MY |
| Total nitrogen (g·kg^-1^) | | 2.20 | 2.40 | 3.10 |
| Total phosphorus (g·kg^-1^) | | 2.50 | 1.70 | 2.00 |
| Total potassium (g·kg^-1^) | | 24.40 | 18.10 | 21.10 |
| Alkali-hydrolyzed nitrogen (mg·kg^-1^) | | 120.00 | 105.00 | 124.00 |
| Available phosphorus (mg·kg^-1^) | | 27.60 | 20.30 | 26.10 |
| Available potassium (mg·kg^-1^) | | 290.00 | 244.00 | 258.00 |
| Organic matter (g·kg^-1^) | | 34.40 | 34.40 | 50.10 |
| Soil texture | | calcium chestnut soil | clay loam | black calcium soil |

Supplementary TABLE 2 Effect of cropping systems on soil physical properties in different ecological regions

| ecological | cropping | SWC | BD | TP | PH | SOM |
| --- | --- | --- | --- | --- | --- | --- |
| region | system | (%) | (g·cm ^-3^) | (%) |  | (g.kg^-1^) |
| HZ | Y | 14.49±0.35Bbc | 1.23±0.03Aab | 53.71±1.08Aab | 8.36±0.09Aa | 14.18±0.71Cc |
|  | YS | 17.89±0.66Bab | 1.17±0.08Ab | 55.72±2.96Aa | 8.25±0.08Aa | 29.96±1.23Ca |
|  | S | 19.40±2.11Aa | 1.22±0.02Aab | 54.72±0.82Aab | 7.51±0.12Bd | 17.46±0.23Cc |
|  | YJ | 13.68±1.88Bc | 1.19±0.01Aab | 54.97±0.47Aab | 8.01±0.10Ab | 27.10±2.23Cab |
|  | J | 14.78±3.37Bbc | 1.20±0.03Aab | 54.59±1.25Aab | 7.58±0.09Bd | 18.69±1.74Cc |
|  | YC | 17.03±1.01Babc | 1.24±0.06Aab | 53.33±2.10Aab | 7.43±0.06Bd | 25.06±4.36Cb |
|  | C | 16.48±0.67Babc | 1.23±0.02Aa | 51.82±0.71Ab | 7.82±0.07Ac | 17.44±0.16Cc |
| GN | Y | 14.01±0.67Bab | 1.21±0.02Aab | 54.34±0.62Aab | 8.21±0.04Aa | 34.55±0.25Bc |
|  | YS | 14.86±1.61Ca | 1.14±0.03Ab | 56.86±0.99Aa | 8.19±0.10Aa | 37.55±0.11Bb |
|  | S | 13.94±0.72Bab | 1.19±0.03Aab | 55.09±1.23Aab | 7.31±0.08Bc | 35.07±1.72Bc |
|  | YJ | 14.52±0.61Bab | 1.17±0.04Aab | 55.72±1.58Aab | 8.02±0.10Aab | 37.62±0.13Bb |
|  | J | 15.66±0.58Ba | 1.20±0.04Aab | 54.72±1.63Aab | 7.87±0.11Ab | 35.48±0.51Bbc |
|  | YC | 15.54±0.35Ba | 1.23±0.04Aab | 53.58±1.60Ab | 8.13±0.03Ba | 43.39±1.48Ba |
|  | C | 12.82±0.73Cb | 1.25±0.03Aa | 52.83±1.23Ab | 7.93±0.08Ab | 33.82±1.25Bc |
| MY | Y | 19.24±0.72Abc | 1.26±0.02Aabc | 52.45±0.82Aabc | 8.42±0.09Aa | 46.88±2.21Ad |
|  | YS | 21.14±0.41Ab | 1.20±0.03Abc | 54.59±0.94Aab | 7.63±0.03Ad | 55.16±0.90Aa |
|  | S | 18.97±0.62Abc | 1.22±0.04Aabc | 53.96±1.63Aabc | 7.90±0.04Abc | 50.34±0.61Ac |
|  | YJ | 18.44±0.88Ac | 1.20±0.03Abc | 54.59±1.25Aab | 8.04±0.11Ab | 51.20±2.03Abc |
|  | J | 23.43±1.87Aa | 1.18±0.02Ac | 55.35±0.64Aa | 7.86±0.09Ac | 51.64±0.83Abc |
|  | YC | 20.87±1.01Ab | 1.27±0.04Aab | 51.95±1.55Abc | 8.32±0.05Aa | 53.50±0.89Aab |
|  | C | 19.30±0.56Abc | 1.29±0.04Aa | 51.45±1.55Ac | 7.91±0.06Abc | 51.43±0.35Abc |

Note: SWC, soil water content; BD, soil bulk density; TP, total soil porosity; PH, soil acidity and alkalinity; SOM, soil organic matter. The bars show the standard errors. Lowercase letters represent the significant difference within the same ecological regions under different cropping systems, while uppercase letters indicate the significant difference within different ecological regions under the same cropping systems. Significance was employed at 0.05. Ecological regions (HZ, Huangshui Valley; GN, Sanjiangyuan District; MY, Qilian Mountain Basin). Cropping systems (Y, oats unicast; YS, oats and forage peas mixed sowing; S, forage peas unicast; YJ, oats and common vetch mixed sowing; J, common vetch unicast; YC, oats and fava beans mixed sowing; C, fava beans unicast). The same as below.

Supplementary TABLE 3 Effect of cropping systems on soil physical properties in different ecological regions

| Ecological  region | Cropping  system | TN  (g·kg^-1^) | ANN  (mg·kg^-1^) | NN  (mg·kg^-1^) | SON  (mg·kg^-1^) | MBN  (mg·kg^-1^) |
| --- | --- | --- | --- | --- | --- | --- |
| HZ | Y | 1.38±0.03Cd | 2.78±0.16Bbc | 4.79±0.06Cd | 9.60±1.53Ad | 86.18±5.41Ad |
|  | YS | 1.89±0.05Ca | 3.24±0.22Cbc | 5.79±0.25Bc | 15.54±0.86Aa | 114.25±0.25Bb |
|  | S | 1.57±0.04Cc | 4.23±0.21Ba | 7.76±0.21Ba | 10.67±0.86Acd | 110.35±0.19Bbc |
|  | YJ | 1.85±0.05Ca | 3.12±0.40Bbc | 6.30±0.09Bbc | 12.77±1.56Abc | 126.10±0.07Aa |
|  | J | 1.58±0.06Cbc | 3.60±0.54Bab | 6.44±0.58Bb | 11.87±0.03Abcd | 103.20±8.10Ac |
|  | YC | 1.69±0.07Cb | 2.91±0.24Bc | 5.04±0.18Cd | 13.58±1.59Aab | 110.40±0.49Bbc |
|  | C | 1.65±0.05Cbc | 3.33±0.29Cbc | 6.81±0.28Cb | 11.65±0.45Abcd | 91.20±0.12Cd |
| GN | Y | 2.38±0.10Bc | 3.02±0.18Bd | 5.87±0.10Bd | 8.57±1.78Ac | 99.23±3.58Ad |
|  | YS | 2.64±0.03Bb | 3.88±0.08Bb | 6.19±0.19Bd | 13.40±1.23Aab | 115.22±1.27Bc |
|  | S | 2.63±0.07Bb | 4.44±0.41Ba | 8.46±0.47Ba | 11.62±0.43Ab | 96.72±0.21Cd |
|  | YJ | 2.67±0.07Bab | 3.67±0.20ABc | 6.54±0.22Bcd | 11.72±0.43Ab | 115.35±0.78Bc |
|  | J | 2.53±0.07Bbc | 3.90±0.08Bb | 7.38±0.61Bb | 9.41±0.02Cc | 123.80±0.16Bb |
|  | YC | 2.81±0.06Ba | 3.34±0.26Bcd | 7.10±0.12Bbc | 14.01±0.04Aa | 139.23±0.11Aa |
|  | C | 2.55±0.11Bb | 4.65±0.17Ba | 8.80±0.08Ba | 9.74±0.02Bc | 122.76±0.27Bb |
| MY | Y | 2.79±0.23Ac | 4.61±0.29Ac | 6.70±0.15Cd | 10.65±0.29Ad | 106.57±13.24Ad |
|  | YS | 3.57±0.03Aa | 5.03±0.10Abc | 8.68±0.04Ac | 14.91±0.02Aa | 147.33±0.50Aa |
|  | S | 3.32±0.17Aab | 5.91±0.28Aa | 11.99±0.32Aa | 11.71±0.21Ac | 125.43±3.62Abc |
|  | YJ | 3.53±0.06Aa | 4.67±0.58Ac | 7.23±0.25Ad | 13.35±0.05Ab | 132.27±5.47Ab |
|  | J | 3.13±0.04Ab | 4.86±0.08Abc | 9.74±0.41Ab | 11.30±0.27Bcd | 115.57±0.21Bcd |
|  | YC | 3.55±0.02Aa | 4.79±0.16Ac | 8.74±0.33Ac | 14.35±0.05Aa | 136.30±3.91Aab |
|  | C | 3.49±0.07Aa | 5.54±0.34Aab | 10.41±0.47Ab | 11.27±0.78Acd | 124.80±0.24Abc |

Supplementary TABLE 4 Effect of cropping systems on soil physical properties in different ecological regions

| Ecological  region | Cropping  system | NR  (mg·kg^-1^·24h^-1^) | ALPT  (mg·kg^-1^·24h^-1^) | UE  (mg·kg^-1^·24h^-1^) | CAT  (mg·kg^-1^·24h^-1^) | SC  (mg·kg^-1^·24h^-1^) |
| --- | --- | --- | --- | --- | --- | --- |
| HZ | Y | 0.16±0.01Bd | 0.45±0.01Ba | 0.73±0.01Ad | 1.56±0.162Bc | 10.10±0.05Ac |
|  | YS | 0.26±0.01Ba | 0.41±0.02Bc | 1.21±0.11Ba | 2.37±0.01Ba | 13.39±2.54Aab |
|  | S | 0.19±0.01Bc | 0.43±0.01Bbc | 0.81±0.04Bcd | 1.72±0.05Cbc | 11.19±0.33Abc |
|  | YJ | 0.22±0.02Bb | 0.47±0.01Ba | 1.14±0.09Bab | 2.22±0.02Ca | 13.65±0.06Aa |
|  | J | 0.20±0.01Bc | 0.42±0.01Cc | 0.83±0.05Bcd | 1.97±0.20Bb | 10.79±0.17Ac |
|  | YC | 0.25±0.01Ba | 0.46±0.03Ba | 1.08±0.07Bab | 2.25±0.01Ba | 11.49±0.41Babc |
|  | C | 0.18±0.01Bc | 0.43±0.01Bc | 0.99±0.09Bbc | 1.88±0.09Bb | 11.32±0.23Abc |
| GN | Y | 0.21±0.02Ab | 0.49±0.01Bab | 0.89±0.20Ac | 2.14±0.09Ade | 9.08±0.11Ad |
|  | YS | 0.27±0.01Ba | 0.42±0.01Bd | 1.32±0.08ABab | 0.95±0.00Bd | 13.28±0.23Ab |
|  | S | 0.22±0.01Ab | 0.46±0.02Abc | 1.11±0.114Abc | 2.30±0.02Acd | 11.43±0.10Ac |
|  | YJ | 0.26±0.01Aa | 0.48±0.02Bab | 1.29±0.13ABab | 2.66±0.04Aab | 14.06±0.78Aab |
|  | J | 0.22±0.01Bb | 0.45±0.01Bcd | 0.94±0.06ABc | 2.32±0.14ABcd | 10.39±1.26Acd |
|  | YC | 0.28±0.01Aa | 0.51±0.02ABa | 1.36±0.05Aa | 2.78±0.17Aa | 15.78±0.65Aa |
|  | C | 0.23±0.00Ab | 0.44±0.01ABcd | 0.93±0.05Bc | 1.90±0.13Be | 9.87±1.55Ad |
| MY | Y | 0.19±0.01Ad | 0.54±0.01Aa | 0.92±0.05Ad | 1.99±0.17Ac | 10.14±0.72Ad |
|  | YS | 0.30±0.01Aa | 0.47±0.01Ad | 1.43±0.04Aa | 2.84±0.04Aa | 16.83±1.62Aa |
|  | S | 0.23±0.00Ac | 0.49±0.01Acd | 1.19±0.07Ab | 2.06±0.13Bc | 10.38±0.98Ad |
|  | YJ | 0.27±0.01Ab | 0.54±0.01Aa | 1.39±0.02Aa | 2.38±0.09Bb | 13.07±0.03Abc |
|  | J | 0.20±0.01Ad | 0.48±0.01Acd | 1.07±0.05Ac | 2.50±0.21Ab | 12.04±0.14Acd |
|  | YC | 0.28±0.01Ab | 0.52±0.01Aab | 1.38±0.04Aa | 2.66±0.08Aab | 14.78±0.84Ab |
|  | C | 0.23±0.01Ac | 0.50±0.01Abc | 1.21±0.06Ab | 2.43±0.16Ab | 10.36±0.08Ad |


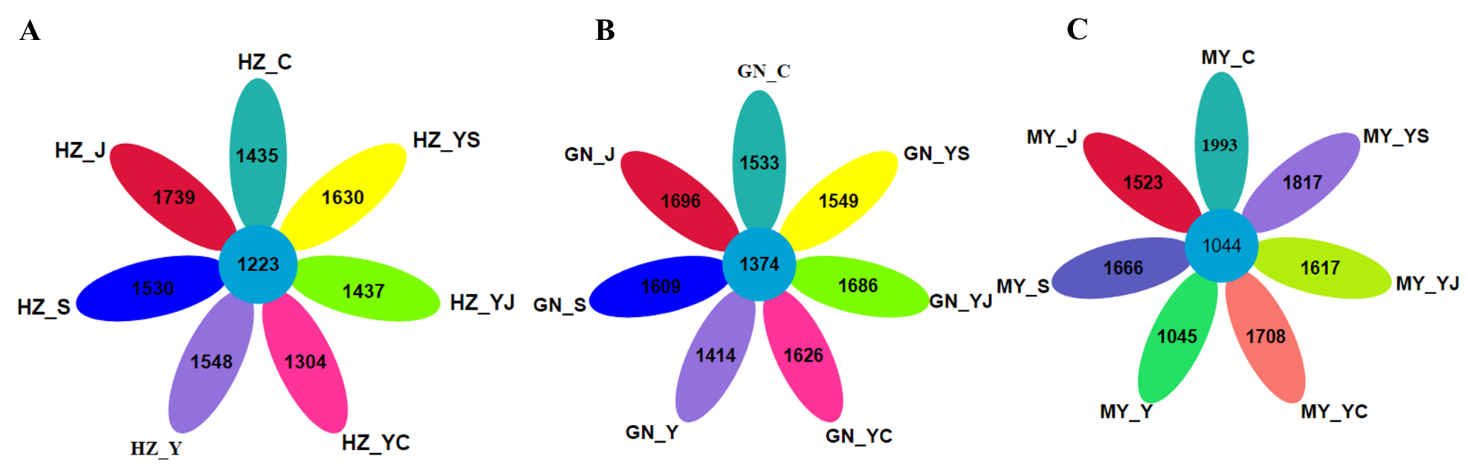


Supplementary FIGURE 1 Bacterial venn diagrams of different ecological regions and different cropping systems. A, Number of unique and common OTUs in all implant systems in HZ; B, Number of unique and common OTUs in all implant systems in GN; C, Number of unique and common OTUs in all implant systems in GN.


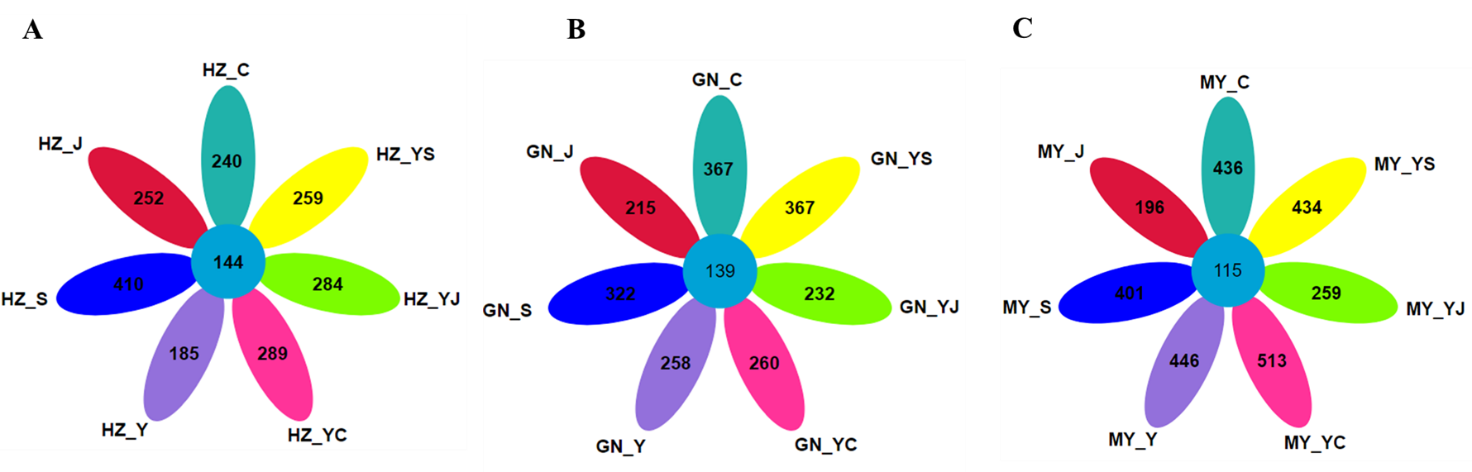


Supplementary FIGURE 2 Fungi venn diagrams of different ecological regions and different cropping systems. A, Number of unique and common OTUs in all implant systems in HZ; B, Number of unique and common OTUs in all implant systems in GN; C, Number of unique and common OTUs in all implant systems in GN.

**Reference**

Abdelmagid, H.M., and Tabatabai, M.A. (1987). Nitrate reductase activity of soils. *Soil Biology & Biochemistry* 19(4)**,** 421-427.

Chen, C.R., Xu, Z.H., Keay, P., and Zhang, S.L. (2005). Total soluble nitrogen in forest soils as determined by persulfate oxidation and by high temperature catalytic oxidation. *Australian Journal of Soil Research* 43(4)**,** 515-523.

Chen, F.S., Zeng, D.H., Fahey, T.J., and Liao, P.F. (2010). Organic carbon in soil physical fractions under different-aged plantations of Mongolian pine in semi-arid region of Northeast China. *Applied Soil Ecology* 44(1)**,** 42-48.

Crepin, J., and Johnson, L.R. (1993). Soil Sampling and Methods of Analysis. *Journal of Environmental Quality* 38(1)**,** 15-24.

Joergensen, R.G. (1996). The fumigation-extraction method to estimate soil microbial biomass: Calibration of the kEC value. *Soil Biology & Biochemistry* 28(1)**,** 25-31.

Kandeler, E., and Gerber, H. (1988). Short-term assay of soil urease activity using colorimetric determination of ammonium. *Biology & Fertility of Soils* 6(1)**,** 68-72.

Liu, J., Bergkvist, G.R., and Ulén, B. (2015). Biomass production and phosphorus retention by catch crops on clayey soils in southern and central Sweden. *Field Crops Research* 171(171)**,** 130-137.

Wang, L., Kaur, M., Zhang, P., Li, J., and Xu, M. (2021). Effect of Different Agricultural Farming Practices on Microbial Biomass and Enzyme Activities of Celery Growing Field Soil. *Int J Environ Res Public Health* 18(23). doi: 10.3390/ijerph182312862.
